# Supplementary material for: Treatment of Intervertebral Disk Disease by the Administration of mRNA Encoding a Cartilage-Anabolic Transcription Factor
Source: Mol Ther Nucleic Acids. 2019 Feb 22;16:162–71. doi: 10.1016/j.omtn.2019.02.012 (PMC6424144; doi:10.1016/j.omtn.2019.02.012)
Supplement: Document S1. Figures S1–S9 [file mmc1.pdf]

OMTN, Volume *16*

Supplemental Information

Treatment of Intervertebral Disk Disease by the Administration of mRNA Encoding a Cartilage-Anabolic Transcription Factor

Chin-Yu Lin, Samuel Thomas Crowley, Satoshi Uchida, Yuji Komaki, Kazunori Kataoka, and Keiji Itaka

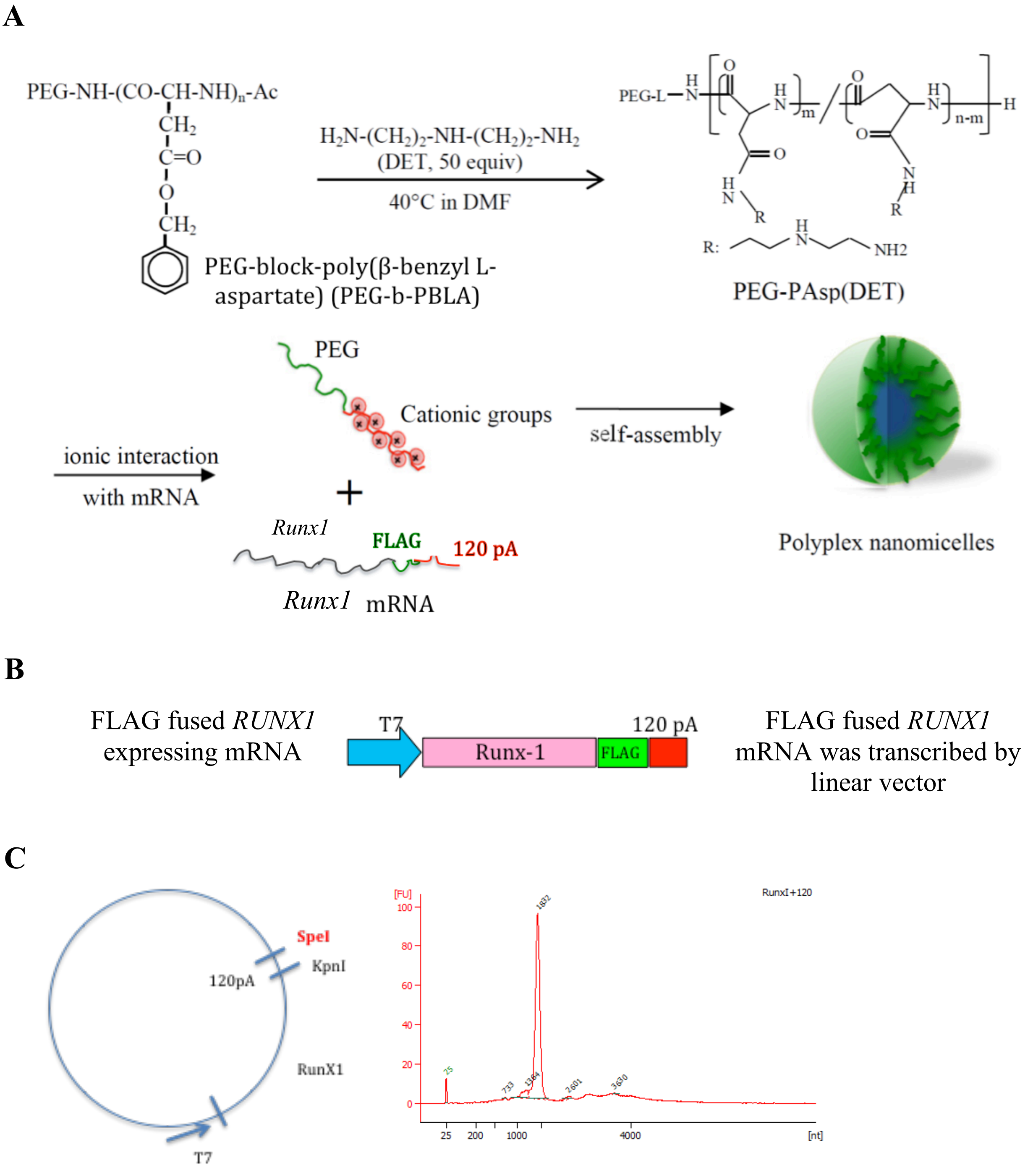

Fig. S1. (A) PEG- Poly{N-[N'-(2-aminoethyl)-2-aminoethyl]aspartamide} block copolymer (PEG-PAsp[DET]) was synthesized and self-assembled with mRNA to form polyplex nanomicelles. (B) The Runx1-FL sequence was sub-cloned into a pSP73 vector, containing a 120 bp chemically synthesized poly(d(A/T) fragment downstream of the cDNA region. (C) The vectors were linearized with SpeI for IVT, and all transcribed mRNAs were analyzed for size and purity with the Agilent RNA 6000 Nano Assay on a BioAnalyzer 2100.

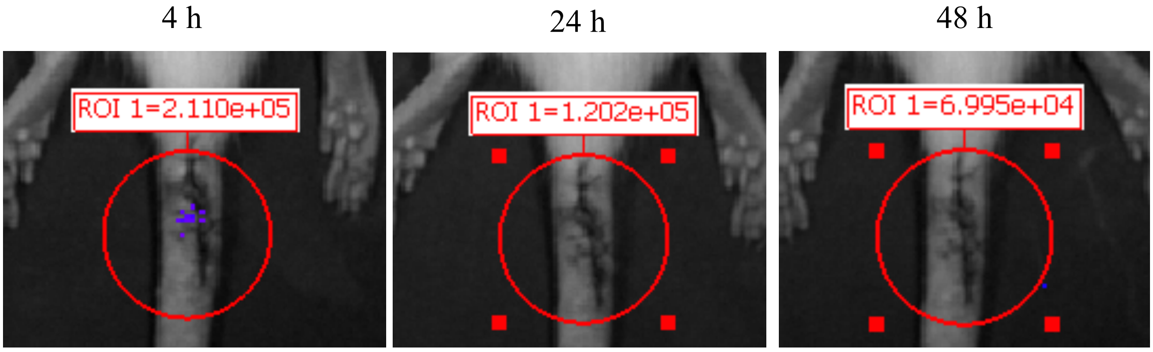

Fig. S2. IVIS images of *Luc* mRNA in the form of naked mRNA without nanomicelles.

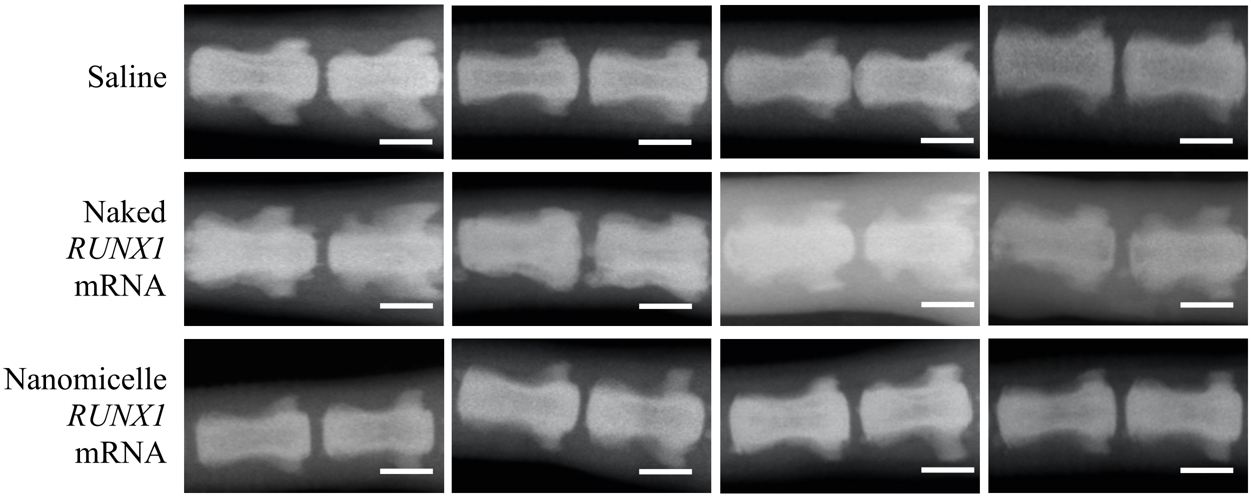

Fig. S3. All other radiographic images of coccygeal disk at 4 weeks post-injection (bar= 4 mm).

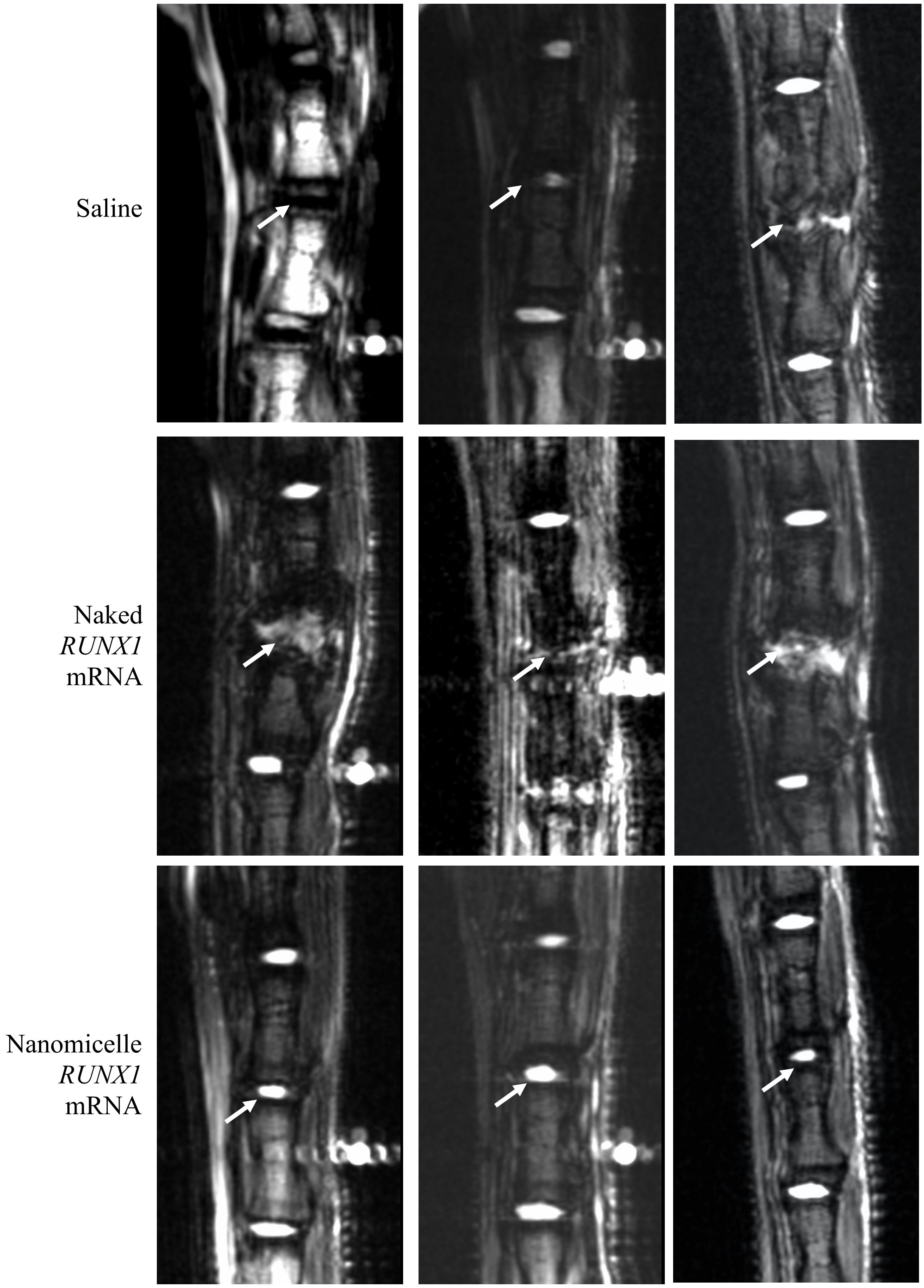

Fig. S4. All other MRI-T2 images of coccygeal disk at 4 weeks post-injection. Arrows indicate the micro-injected disks.

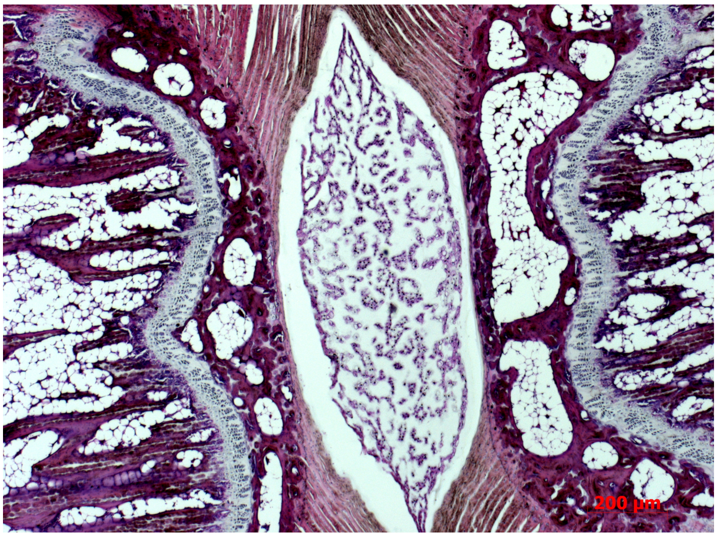

Fig. S5. H&E staining of Intact disk.

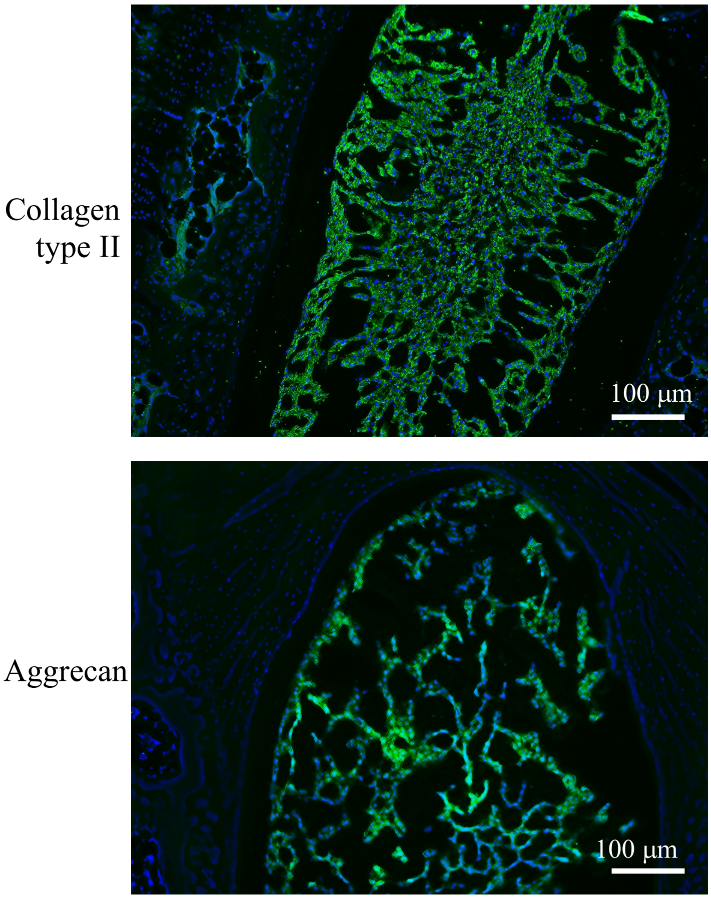

Fig. S6. Type II Collagen and aggrecan IHC of Intact disk.

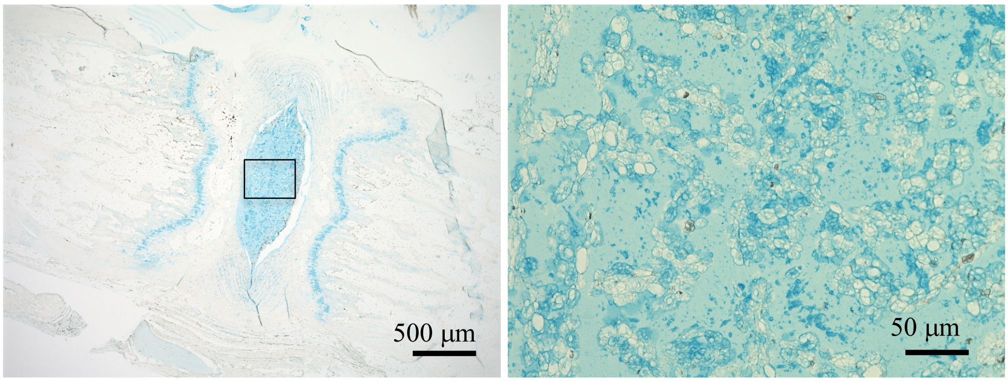

Fig. S7. Alcian blue staining of intact disk.

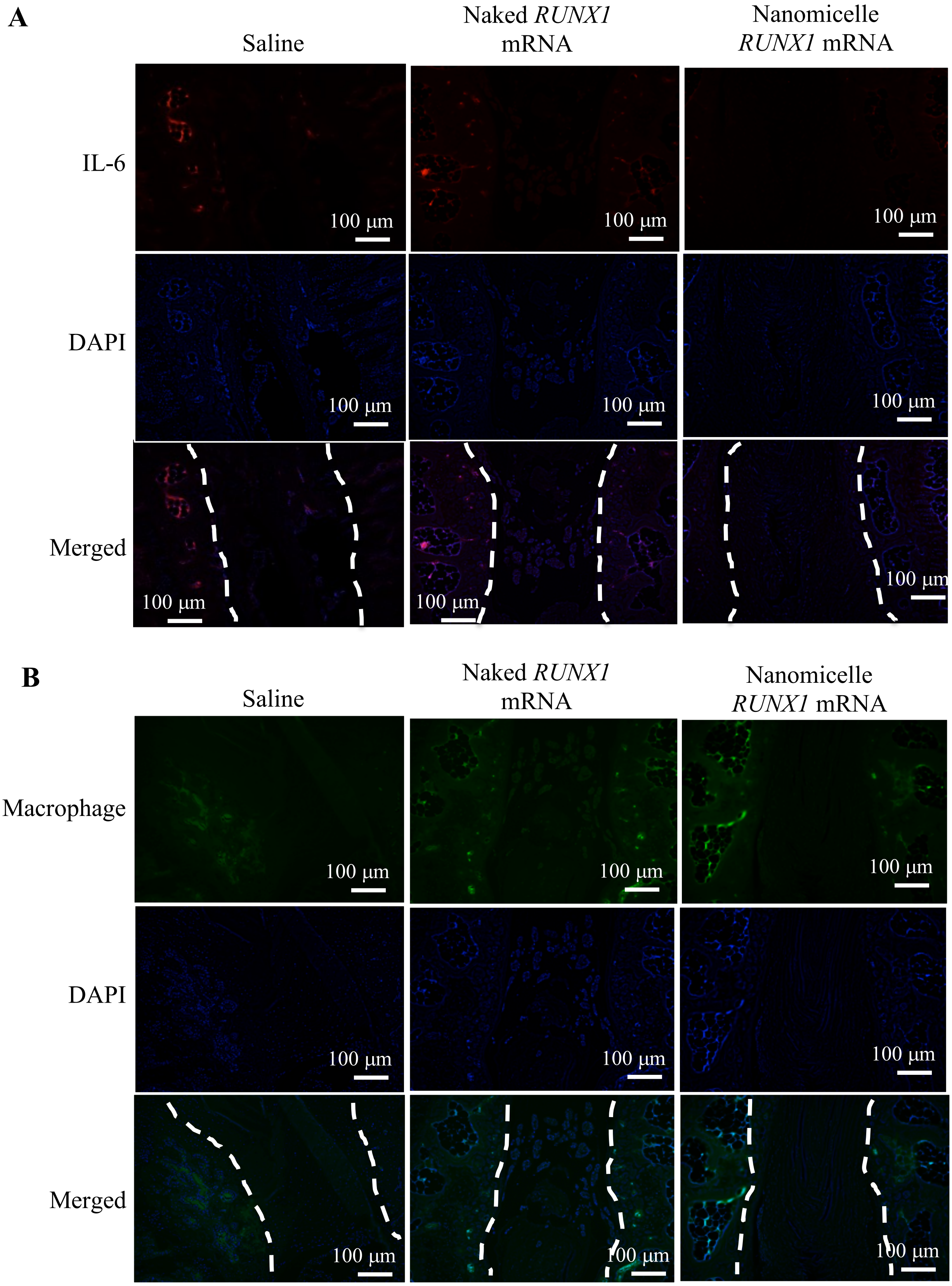

Fig. S8. **IL-6 and macrophage IHC staining of disks removed at 2 weeks post-injection. (A)** IL-6 IHC, the 2nd Ab was labeled with Alexa-647. **(B)** Macrophage IHC, the 2nd Ab was labeled with Alexa-488. Dotted lines indicate the disk area. Bar = 100 μm.

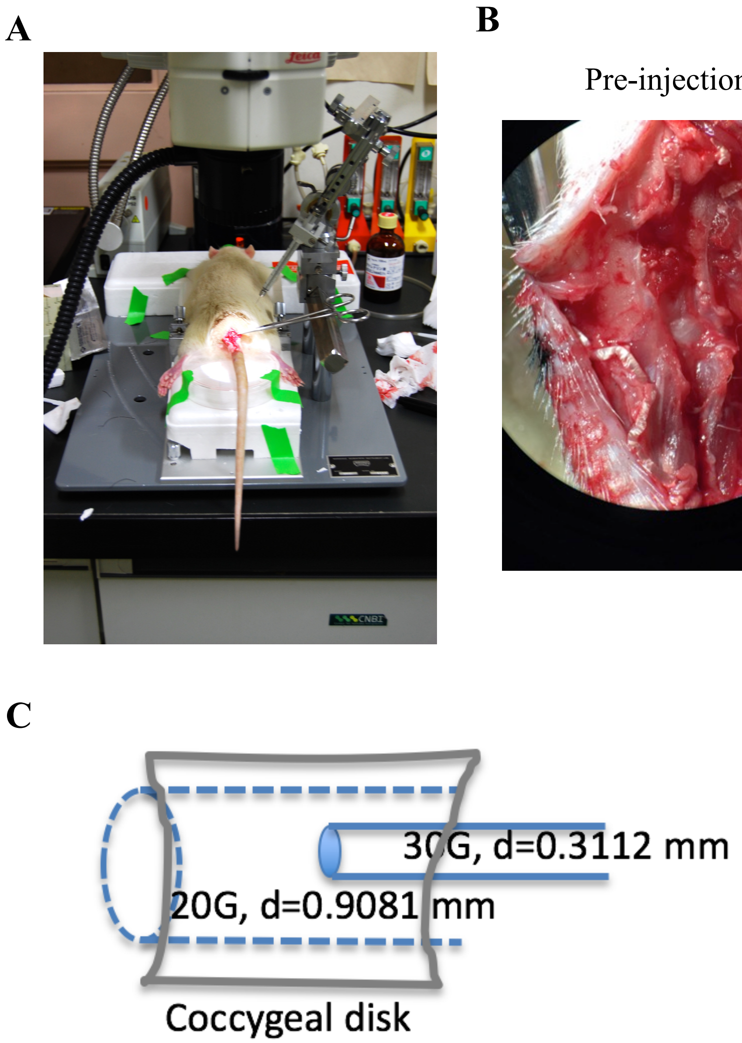

Fig. S9. (A) Rat coccygeal disks were operated on under the assistance of a stereotaxic instrument and microscope. (B) Gene mixtures were micro-injected with 30G micro-injection needle at 1 μl/min at a 1 mm depth. (C) Illustration of disk punctured with 20G needle, subsequently micro-injected with 30G needle through the larger track.
