# Supplementary material for: Treatment of Intervertebral Disk Disease by the Administration of mRNA Encoding a Cartilage-Anabolic Transcription Factor
Source: Mol Ther Nucleic Acids. 2019 Feb 22;16:162–71. doi: 10.1016/j.omtn.2019.02.012 (PMC6424144; doi:10.1016/j.omtn.2019.02.012)
Supplement: Document S2. Article plus Supplemental Information [file mmc2.pdf]

Molecular Therapy
Nucleic Acids
Original Article

Treatment of Intervertebral Disk Disease by the Administration of mRNA Encoding a Cartilage-Anabolic Transcription Factor

Chin-Yu Lin,[1,2] Samuel Thomas Crowley,[2,3] Satoshi Uchida,[2,4] Yuji Komaki,[5] Kazunori Kataoka,[2,6] and Keiji Itaka[2,3]

[1]Institute of New Drug Development, China Medical University, Taichung 40402, Taiwan; [2]Innovation Center of NanoMedicine, Kawasaki Institute of Industrial Promotion, Kawasaki, Kanagawa 210-0821, Japan; [3]Department of Biofunction Research, Institute of Biomaterials and Bioengineering, Tokyo Medical and Dental University (TMDU), Chiyoda-ku, Tokyo 101-0062, Japan; [4]Department of Bioengineering, Graduate School of Engineering, The University of Tokyo, Bunkyo, Tokyo 113-8656, Japan; [5]Central Institute for Experimental Animals, Kawasaki, Kanagawa 210-0821, Japan; [6]Policy Alternatives Research Institute, The University of Tokyo, Bunkyo, Tokyo 113-0033, Japan

Intervertebral disk (IVD) degeneration is often associated with severity of lower back pain. IVD core is an avascular, highly hydrated tissue composed of type II collagen, glycosaminoglycans, and proteoglycans. The disk degeneration is not only a destruction of IVD structure but also is related to a disorder of the turnover of the disk matrix, leading the jelly-like IVD core to be replaced by fibrous components. Here we present a disease-modifying strategy for IVD degenerative diseases by direct regulation of the cells in the IVD using mRNA medicine, to alter the misbalanced homeostasis during disk degeneration. When mRNA encoding a cartilage-anabolic transcription factor, runt-related transcription factor-1, was administered to a rat model of coccygeal disk degeneration using a polyplex nanomicelle composed of polyethylene glycol-polyamino acid block copolymers and mRNA, the disk height was maintained to a significantly higher extent (≈81%) compared to saline control (69%), with prevention of fibrosis in the disk tissue. In addition, the use of nanomicelles effectively prevented inflammation, which was observed by injection of naked mRNA into the disk. This proof-of-concept study revealed that mRNA medicine has a potential for treating IVD degenerative diseases by introducing a cartilage-anabolic factor into the host cells, proposing a new therapeutic strategy using mRNA medicine.

INTRODUCTION

Lower back and neck pain are frequently associated with intervertebral disk (IVD) degeneration, and imaging studies have found an association between prevalent IVD degeneration and severity of lower back pain.[1,2] The IVD is composed of a central hydrophilic proteoglycan-rich gelatinous core, the nucleus pulposus (NP), which is surrounded by a multilamellar collagenous ring, the annulus fibrosus, and cartilaginous and bony endplates that separate the disk from the vertebrae.[3] The IVD core is an avascular, highly hydrated tissue due to the extracellular matrix (ECM) surrounding nucleus pulposus cells, which are composed of type II collagen, glycosaminoglycans (GAGs), and proteoglycans such as aggrecan.[4,5] The IVD is a dynamic tissue, in which the ECM undergoes continuous turnover.[6] Disk disease is not only a destruction of IVD structure but also is related to a disorder of the turnover, due to the shortage of factors involved in the homeostasis of the disk matrix.[7] Even when anabolic and catabolic activities of cells in the IVD are normally maintained, excessive mechanical stress or other stimuli can alter the homeostasis and initiate the degenerative cascade.

Current approaches to prevent IVD degeneration are limited. Most treatments are only symptomatic, and there is no treatment that has been proven to directly regulate homeostasis in the IVD.[8] It is apparent that removal of NP tissue during discectomy accelerates the degeneration of the IVD after the surgery.[9] Thus, a novel approach aiming at the restoration of IVD homeostasis is strongly demanded.

In this context, we propose mRNA as a means to regulate IVD cells, which could alter the misbalanced homeostasis during disk degeneration. This idea is based on our previous study to prevent cartilage degeneration in the joints of osteoarthritis model mice, where the chondrocytes in the joint cartilage were modified by introducing a cartilage-anabolic factor, runt-related transcription factor-1 (RUNX1), via intraarticular administration of RUNX1-encoding mRNA.[10,11] The modified chondrocytes showed an increase in the expression of cartilage-anabolic markers and proliferation, resulting in the augmentation of the net resistance of the articular cartilage to the mechanical loading. For IVD, especially for the core where the components such as type II collagen, GAGs, and proteoglycans are similar to those in the hyaline cartilage in the joint, *RUNX1* introduction into NP cells in the IVD core would be expected to induce

Received 6 January 2019; accepted 16 February 2019;
https://doi.org/10.1016/j.omtn.2019.02.012.

Correspondence: Keiji Itaka, Department of Biofunction Research, Institute of Biomaterials and Bioengineering, Tokyo Medical and Dental University (TMDU), 2-3-10 Kanda-Surugadai, Chiyoda-ku, Tokyo 101-0062, Japan.
E-mail: itaka.bif@tmd.ac.jp

This is an open access article under the CC BY license (http://creativecommons.org/licenses/by/4.0/).

mucoid material production, maintain the hydration content in the core, and be effective for decelerating the disk degeneration.

The use of mRNA for administering transcription factor(s) is a key factor for the therapeutic strategy. It is an alternative to gene therapy using viral and non-viral vectors,[12,13] but there are many advantages of mRNA over the vectors. There is no requirement for nuclear entry, which improves the transfection efficiency in slowly dividing or non-dividing primary cells, such as neural cells and chondrocytes[11,14,15] and, potentially, the NP cells in the IVD. Transient protein translation with negligible risk of random integration into the genome are important factors for the treatment of disk diseases, because the uncontrolled expression of intracellular signal protein(s) by gene vectors might cause hazardous side effects. Such risks will not be accepted for the non-lethal degenerative diseases.

In this study, we used a rat model of coccygeal disk degeneration induced by needle puncture.[16–18] It is hypothesized that the introduction of *RUNX1* mRNA would stimulate mucoid material secretion from resident NP cells to maintain the hydration content and decelerate disk degeneration processes. A nanocarrier composed of polyethylene glycol (PEG)-polyamino acid (Poly{N-[N'-(2-aminoethyl)-2-aminoethyl]aspartamide}) block copolymers (PEG-PAsp [DET]) and mRNA, called polyplex nanomicelles, was used for introducing the mRNA into the disk.[11,15,19–21] The nanomicelles are based on self-assembly of the block copolymers, possessing a PEG outer layer and mRNA-containing core for stable retention of the mRNA in the nanomicelles. In addition, the PEG shielding on the carrier surface effectively prevented the inflammatory responses that are often caused by unfavorable immunogenicity of mRNA. After receiving *RUNX1* mRNA using the nanomicelles, or by the form of naked mRNA, the disk was analyzed morphologically by X-ray, qualitatively using MRI-T2 imaging to measure hydration content, and immunohistologically to evaluate the RUNX1 expression and extracellular matrix composition. This study provides a proof of concept of a new therapeutic for treating disk degeneration diseases by the direct administration of mRNA into the disk.

RESULTS

Evaluation of Protein Expression from Administered mRNA into Rat Coccygeal Disk

In this study, block copolymers of PEG and Poly{N-[N'-(2-aminoethyl)-2-aminoethyl]aspartamide}, PEG-PAsp[DET], were manufactured as mRNA delivery nanocarriers, based on self-assembly through electrostatic interaction with mRNA to form polyplex nanomicelles (Figure S1A). To evaluate the protein expression from the mRNA administered into rat coccygeal disks in quantitative and time-dependent manners, an mRNA encoding luciferase was used. The rat coccygeal 4-5 disk was punctured with a 20G needle, and subsequently it was micro-injected with 2 μg mRNA loaded into nanomicelles in a total volume of 6 μL solution. The luciferase expression was clearly observed by IVIS bioluminescent imaging for 168 h after *Luc* mRNA administration, although expression levels decreased over time (Figures 1A and 1B) (n = 4). When administering the same *Luc* mRNA in the form of naked mRNA without nanomicelles, no luminescence signals were detected on the IVIS images (Figure S2).

To confirm the expression of RUNX1 protein in the disk when *Runx1* mRNA was administered, we evaluated a FLAG peptide that was added to the sequence of *RUNX1* for monitoring RUNX1 expression. By immunohistochemical staining using an anti-FLAG antibody, widely distributed FLAG signals were observed in the disk 2 weeks after injecting *RUNX1* mRNA using nanomicelles (Figure 1C). The signals were weakened at 4 weeks after administration but still clearly visible. In contrast, no FLAG signals were detected in the group receiving naked *RUNX1* mRNA or saline, which was consistent with the results of luciferase expression (Figure 1). Thus, it was confirmed that mRNA administration into coccygeal disk using nanomicelles could produce encoded proteins in the disk tissue.

RUNX1 mRNA Administration Using Nanomicelles Diminished the Coccygeal Disk Height Shrinkage Rate

A representative finding in disk degeneration is the decrease in the disk height, due to loss of hydration content inside the disk core and loss of mechanical strength provided by disk core elasticity. Here, the post-puncture changes in rat coccygeal disk height were examined by X-ray imaging. A pre-puncture image of coccygeal (co)4-5 was taken, then the disk was punctured using a 20G needle. The punctured disk was micro-injected with *RUNX1* mRNA-loaded nanomicelles, naked *RUNX1* mRNA, or saline control using a 30G syringe. An additional X-ray image was taken immediately after the injection as that of 0 week, then images were taken 2 and 4 weeks after the injection.

Representative images from pre-puncture to 4 weeks are presented in Figure 2A. Other images of 4 rats are shown in Figure S3. The changes in disk height were calculated as disk height index (DHI %) with the equation shown in Figure 2B. As shown in Figure 2C, the disk height was slightly decreased immediately after the needle puncture, and it continued to decrease for 4 weeks in all groups. The saline-treated control group showed a decrease in DHI to ≈65% by 2 weeks post-puncture, and it remained after that. The naked *RUNX1* mRNA group showed a decrease in DHI to ≈80% at 2 weeks, and it further declined to ≈75% at 4 weeks. The *RUNX1* mRNA nanomicelle group showed a decrease to ≈87% at 2 weeks and to ≈80% at 4 weeks, and the disk heights were significantly greater than those of saline-treated control at 2 and 4 weeks ($N \geq 5$) ($p < 0.05$ in saline versus *RUNX1* mRNA nanomicelle at 4 weeks). It is suggested that, although *RUNX1* mRNA nanomicelle could not stop the decrease in disk height completely, the nanomicelle was effective for diminishing the disk shrinkage after the puncture.

MRI Evaluation Demonstrated Increased Hydration Content in the Coccygeal Disk after Administration of *RUNX1* mRNA Nanomicelles

The disk shrinkage is considered to be closely related to decreased mucoid materials in the disk center, represented by the decrease in hydration content in the disk core. To analyze the hydration content in

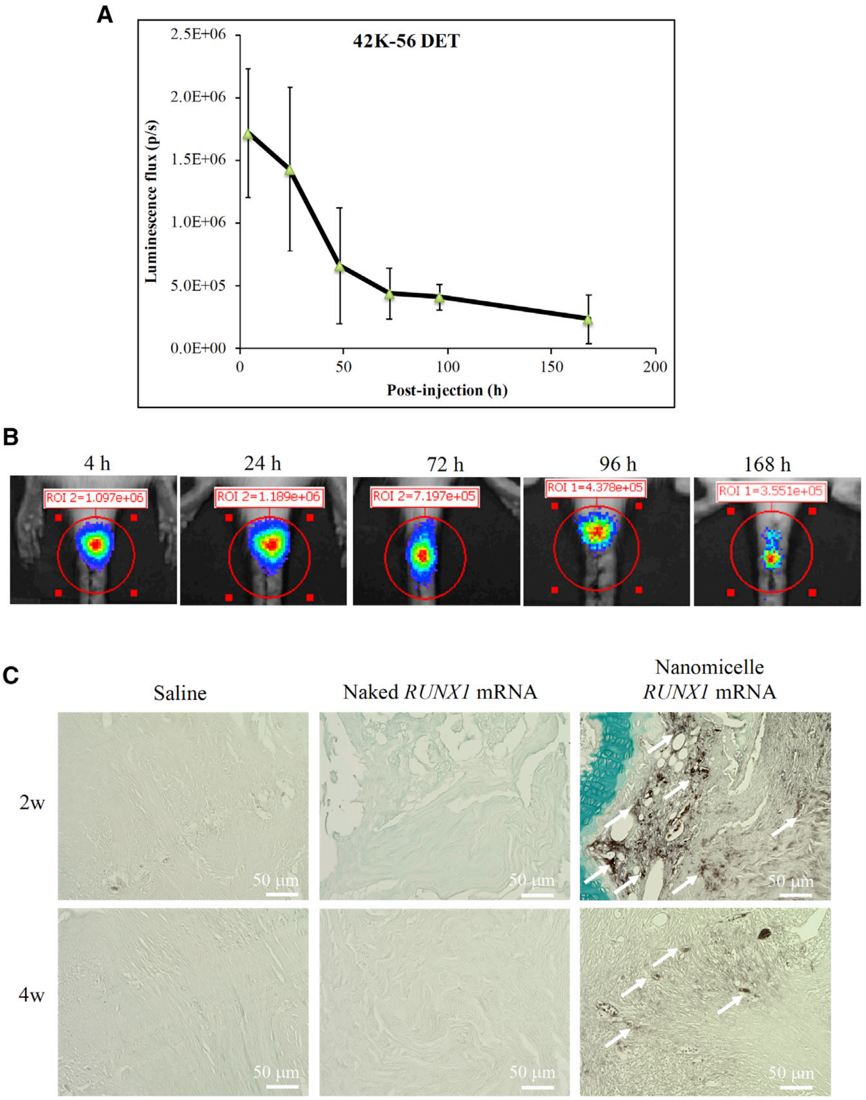

Figure 1. Protein Expression in Rat Coccygeal Disk after the Administration of mRNA Using Polyplex Nanomicelles

(A) Time course of luciferase expression after the administration of luciferase mRNA (*Luc* mRNA), evaluated by IVIS images until 168 h after the administration. Data are expressed as the mean ± SD (n = 4). (B) Representative IVIS images of rat coccygeal disk after *Luc* mRNA administration. (C) Immunohistochemical staining using an anti-FLAG antibody on the sections of coccygeal disks after the administration of *RUNX1* mRNA containing FLAG sequence. Arrows indicate the post-DAB signals.

the disks, MRI examination was also performed on the same rats as in Figure 2. As shown in T2-weighted images (Figure 3) (N ≥ 4), the saline control group showed an almost complete loss of hydration content in the punctured disk, represented by the low signals in the disks, compared with adjacent disks after 2 weeks. In contrast, the *RUNX1* mRNA nanomicelle group showed partial retention of high signals in the disks comparable to other intact disks, indicating the alleviation of disk damage for retaining the hydration content. Interestingly, the naked *RUNX1* mRNA group showed increases in the signals not only in the disk but also in the surrounding tissues, with an irregular appearance. Other MRI images of 4 weeks are presented in Figure S4.

RUNX1 mRNA Nanomicelle Administration Alleviated the Loss of NP Jelly-like Materials

In addition to evaluating disk shrinkage and changes in hydration content, the coccygeal disks were removed for histological examination. H&E staining of coccygeal disks at 4 weeks post-puncture revealed that the control saline injection group showed squeezed disks, with fibrous tissues filling in the disk space (Figure 4). A similar finding of filling by fibrous tissues was observed for the naked mRNA group, with increased cell infiltration into the disk tissues. Together with the MRI findings in Figure 3, it is suggested that the naked mRNA induced local inflammation in the disk, and the details are analyzed later (Figure 7). In contrast, for the *RUNX1* mRNA nanomicelle group, transparent jelly-like materials still existed in the disk core, and some NP-like cells remained, although the fibrous tissues partially infiltrated into the disk space. In comparison with the intact disk (Figure S5), it is suggested that the *RUNX1* mRNA nanomicelle group effectively alleviated the loss of contents in the disk core.

RUNX1 mRNA Nanomicelles Preserved Cartilaginous Components in the Disk

Next, the cartilaginous components, type II collagen and aggrecan, were analyzed in the disks by immunohistochemical staining. These components play an important role in providing the elasticity of normal disk. In the images of *RUNX1* mRNA nanomicelle group, the signals of type II collagen and aggrecan were well observed (Figure 5), although the appearance was altered from that of the intact disk (Figure S6). However, almost no significant signals of type II collagen or aggrecan were detected in the sections of saline and naked *RUNX1* mRNA groups (Figure 5).

In addition, GAG was evaluated by Alcian blue staining 4 weeks after the administration of *RUNX1* mRNA nanomicelles, naked *RUNX1* mRNA, or saline (Figure 6). The GAG contents represented by Alcian blue-positive staining were greater for the *RUNX1* mRNA nanomicelle group compared with the other two groups, although the appearance of Alcian blue-positive staining was different from that of the intact disk (Figure S7), presumably due to the tissue damage by the puncture. These results suggest that the *RUNX1* mRNA administration using

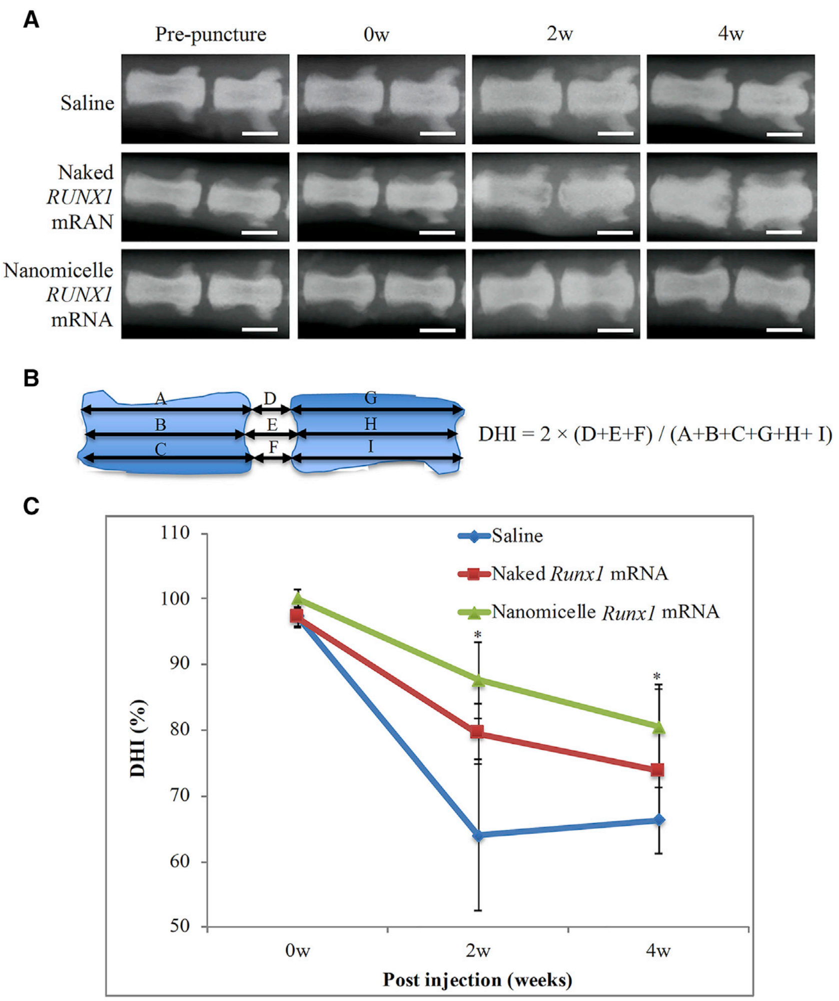

Figure 2. Evaluation of Coccygeal Disk Height by Radiographic Imaging

(A) Representative X-ray images of the coccygeal disk of three groups, injected with *RUNX1* mRNA-loaded nanomicelles, naked *RUNX1* mRNA, and saline. (B) Schematic representation of the method to evaluate disk height by disk height index (DHI). Adjacent vertebral body height and disk height were measured from radiographs using ImageJ. Changes in the DHI of punctured disks are expressed as percentage (DHI % = post-punctured DHI/pre-punctured DHI $\times$ 100%). DHI = 2 $\times$ (D + E + F)/(A + B + C + G + H + I). (C) Changes in DHI % for 4 weeks after the injection (scale bar, 4 mm) ($N \geq 5$). *$p < 0.05$ (saline versus *RUNX1* mRNA nanomicelle).

nanomicelles provided the effect of preserving the cartilaginous components in the disk after disk puncture.

Local Inflammation Induced by *RUNX1* mRNA Was Reduced by Nanomicelles

Finally, the inflammatory responses at the injection site were evaluated, because, as shown in Figure 4, a number of cells might be infiltrated in the disk tissue, especially for the naked *RUNX1* mRNA group. It is known that mRNA administration can elicit a strong immune response through recognition by Toll-like receptor 3 (TLR-3) and TLR-7, inducing secretion of proinflammatory cytokines such as interleukin-6 (IL-6), as well as macrophage infiltration into the lesion. Immunohistochemical analyses for evaluating IL-6 and macrophages were done on the sections of coccygeal disks 2 weeks after mRNA administration; however, no signals were detected for both IL-6 and macrophage on any sections (Figure S8). Then, sections were taken 1 day after the mRNA administration, followed by the immunohistochemical staining. Strong signals of IL-6 and macrophages were observed only for the naked *RUNX1* mRNA group (Figure 7). These results strongly suggest that the naked *RUNX1* mRNA administration produced inflammation due to immunogenicity of the mRNA, leading to macrophage infiltration into the disk as well as IL-6 upregulation. The nanomicelles effectively prevented the inflammatory responses by shielding the mRNA in the nanomicelles.

DISCUSSION

In this study, we demonstrated the therapeutic effects of *RUNX1* mRNA injected into the IVD using nanomicelles. The disk height and hydration content were maintained after disk puncture with prevention of fibrosis in the disk tissue. In addition, the use of nanomicelles effectively prevented inflammation, which was observed by injection of naked mRNA into the disk.

RUNX1 is chosen in this study because RUNX1 is directly involved in Kartogenin-mediated cartilage regeneration and protection, inducing chondrogenesis through regulating the nuclear localization of the core-binding factor beta (CBFβ) transcription complex.[22] RUNX1 also controls type II collagen expression by directly binding with its promoter-responsive elements,[23] and it plays a critical role in controlling mesenchymal stem cell (MSC) differentiation to chondrocytes and their proliferation and survival.[24,25] In addition, RUNX1 would suppress RUNX2 transcription, a master regulator for osteoblast differentiation,[26] inhibiting osteoblast differentiation and terminal chondrocyte hypertrophy.[22] Indeed, these findings of RUNX proteins have been established in the study of joints, but also they have been reported to be closely involved in the pathogenesis of IVD degeneration.[27] Taken together, RUNX1 is a promising candidate for the therapeutic use of disk degeneration by regulating homeostasis in the disk.

The therapeutic effect was most typically presented by MRI images (Figure 3). The T2-high signals in the disk tissues receiving *RUNX1* mRNA nanomicelles clearly indicated retained hydration content compared with other controls 4 weeks after the mRNA administration. In addition, the chondrogenic markers such as GAG, ColII, and aggrecan were generally upregulated (Figures 5 and 6). Therefore,

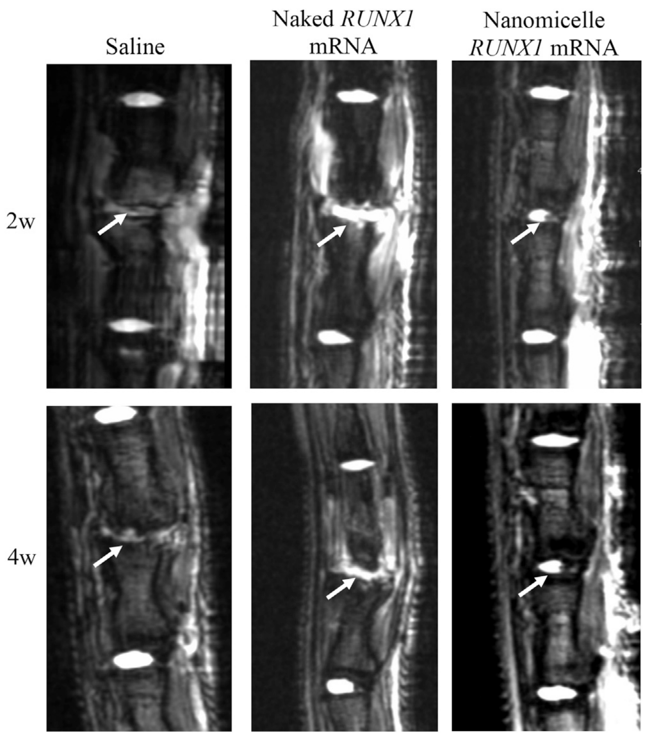

Figure 3. MRI Images of Coccygeal Disk after the Administration of *RUNX1* mRNA

MRI-T2 images 2 or 4 weeks after the injection of *RUNX1* mRNA nanomicelles, naked *RUNX1* mRNA, and saline. Arrows indicate the mRNA-injected disk. Other animals' MRI-T2 images at 4 weeks are shown in Figure S4 (N ≥ 4).

it is reasonable to believe that *RUNX1* mRNA exerted a cartilage-anabolic function in the injured disk tissue, by enhancing the production of hydration content as well as the components that form the disk tissue. We consider the target cells are chiefly the residing cells in the disk tissue, since the RUNX1 expression was confirmed by the FLAG expression in the cells in the disk (Figure 1).

In contrast, the MRI T2-weighted image after injection of naked *RUNX1* mRNA showed the irregular appearance of bright lesions surrounding the injected IVD at 2 and 4 weeks after the mRNA administration (Figure 3). T2-high signals in the disk tissues were apparently higher than those of saline control; however, since almost no RUNX1 expression was obtained by the naked mRNA (Figure 1), the mechanism of maintaining the T2-high signals in the disk tissues should not be the same as that of *RUNX1* mRNA nanomicelles. Otherwise, since a wild-type mRNA is known to be immunogenic via the innate immune system,[28] it is reasonable to assume that the inflammation was induced by the naked mRNA at the IVD lesion and the surrounding tissues, resulting in the irregular appearance with the T2-high signals. Although the acute inflammatory reactions such as macrophage infiltration in the disk with upregulation of IL-6 had disappeared at 2 weeks (Figure S8), the T2-high signals at the lesion might be continued, as observed in the study to treat IVD degeneration using an agent for regulating inflammation.[29] Presumably, the greater disk height of the naked *RUNX1* mRNA group compared with saline control (Figure 2C) might be due to fluid accumulation and disk swelling induced by the local inflammation in the disk.

Eventually, the abnormal findings were not observed for mRNA nanomicelles (Figure 7), clearly demonstrating that the nanomicelles effectively prevented the inflammation. The nanomicelle has a stealth property due to dense PEG palisades on the surface, and the mRNA inside the nanomicelles can be shielded from innate immune system recognition.[21] Unlike mRNA vaccines, which are often administered in the form of naked mRNA, it is apparent that the immunogenic mRNA would be unfavorable for therapeutic purposes. As well as the use of modified mRNA with less immunogenicity, mRNA carriers, such as nanomicelles, are a promising technique for regulating the immune responses in the lesion, even for the case of local administration.

mRNA medicine has great potential for various diseases. Therapeutic effects by *in vivo* mRNA administration were reported so far in

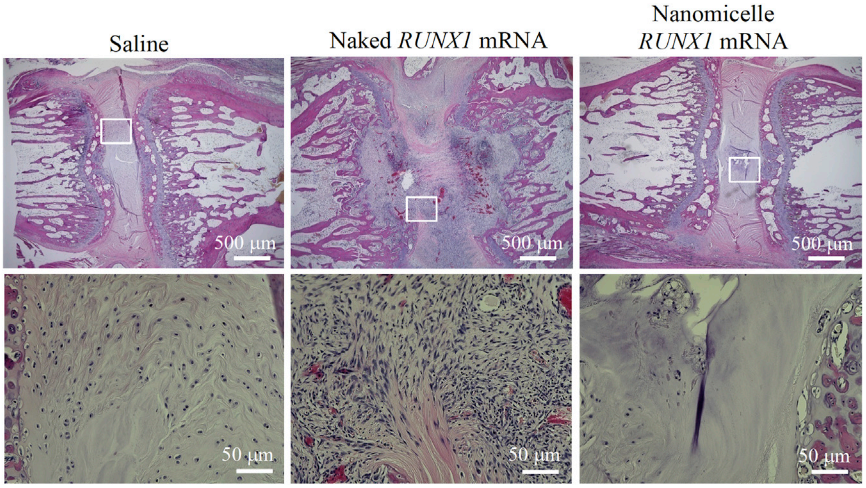

Figure 4. Histological Observation of Coccygeal Disks

Representative H&E images at 4 weeks after the injection of *RUNX1* mRNA nanomicelles, naked *RUNX1* mRNA, and saline. Inset boxes indicate the magnified region shown below each image.

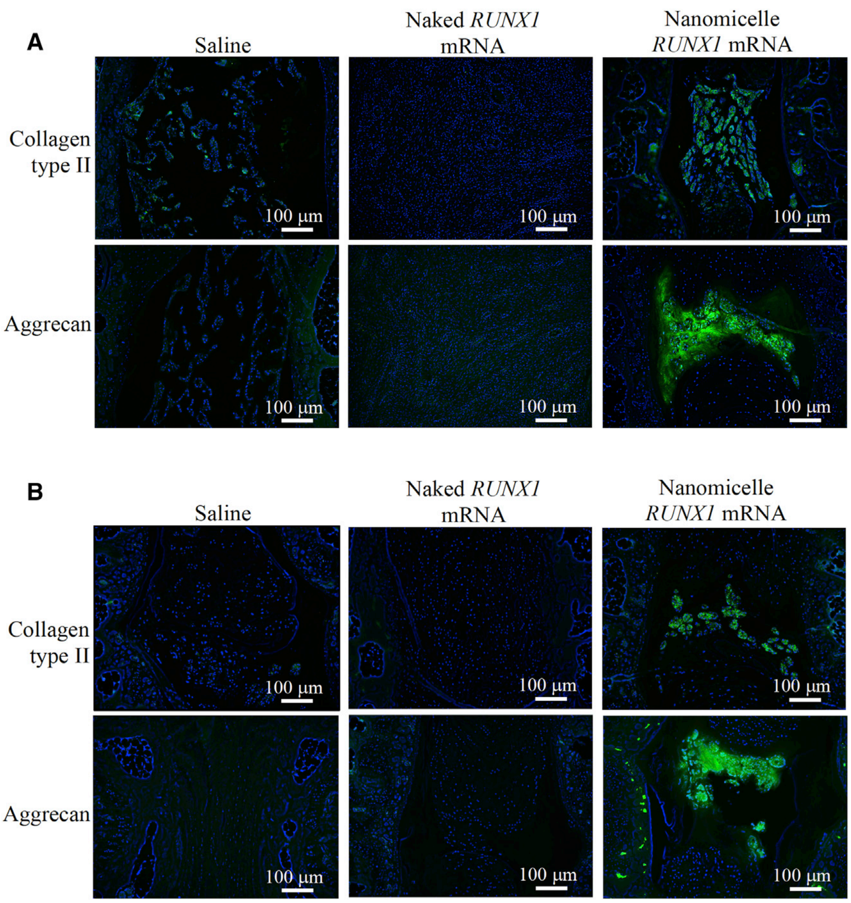

Figure 5. Immunohistochemical Staining of Type II Collagen and Aggrecan

Sections were stained with primary antibodies targeting ColII and Aggrecan, respectively, and further stained with a secondary antibody labeled with Alexa 488. They are shown (A) 2 weeks and (B) 4 weeks after the injection of *RUNX1* mRNA nanomicelles, naked *RUNX1* mRNA, and saline (n = 4). Scale bar, 100 μm.

animal models of cardiac infarction,[30,31] neurologic disorders,[15,20,32,33] hepatitis,[34] genetic diseases,[35,36] and tissue regeneration.[11,37–39] Especially, the administration of transcription factor(s) for directly regulating cell fate and functions can provide a new paradigm for the field of drug development. The conventional processes of drug development, including the screening of candidate molecules, would be unnecessary. Upon identifying the intracellular signals that are related to the physiological or pathological processes of the target diseases, mRNA can be easily used for upregulating the signaling by introducing the key molecule(s). In this regard, the strategy is similar to that of developing nucleic acid medicines such as antisense oligonucleotides and small interfering RNA (siRNA) for gene knockdown. It is apparent that a transient manner of expression is mandatory for the signaling molecules, because continuous activation of specific signaling should cause serious safety concerns. mRNA medicine is likely the only way for this therapeutic approach.

Currently, there are almost no therapies for aborting or slowing the progress of disk degeneration. Tissue engineering by cell transplantation with or without artificial scaffolds is a promising method to preserve the cell metabolism in the disk tissue, and this is now vigorously investigated in many laboratories, some of which have already proceeded to clinical trials.[40–44] However, the preparation of the cells remains a critical issue: it is costly, time consuming, and clinically difficult to obtain sufficient numbers of cells from the elderly patients who often suffer from IVD degenerative diseases. The prevention of disk degeneration using

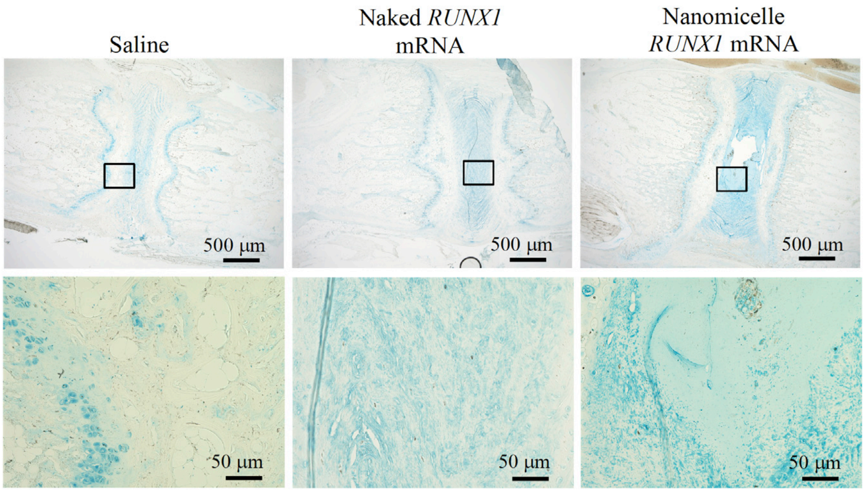

Figure 6. Alcian Blue Staining for Evaluating Glycosaminoglycan

Sections were obtained 4 weeks after the administration of *RUNX1* mRNA nanomicelles, naked *RUNX1* mRNA, and saline. Inset boxes indicate the magnified region shown below each image.

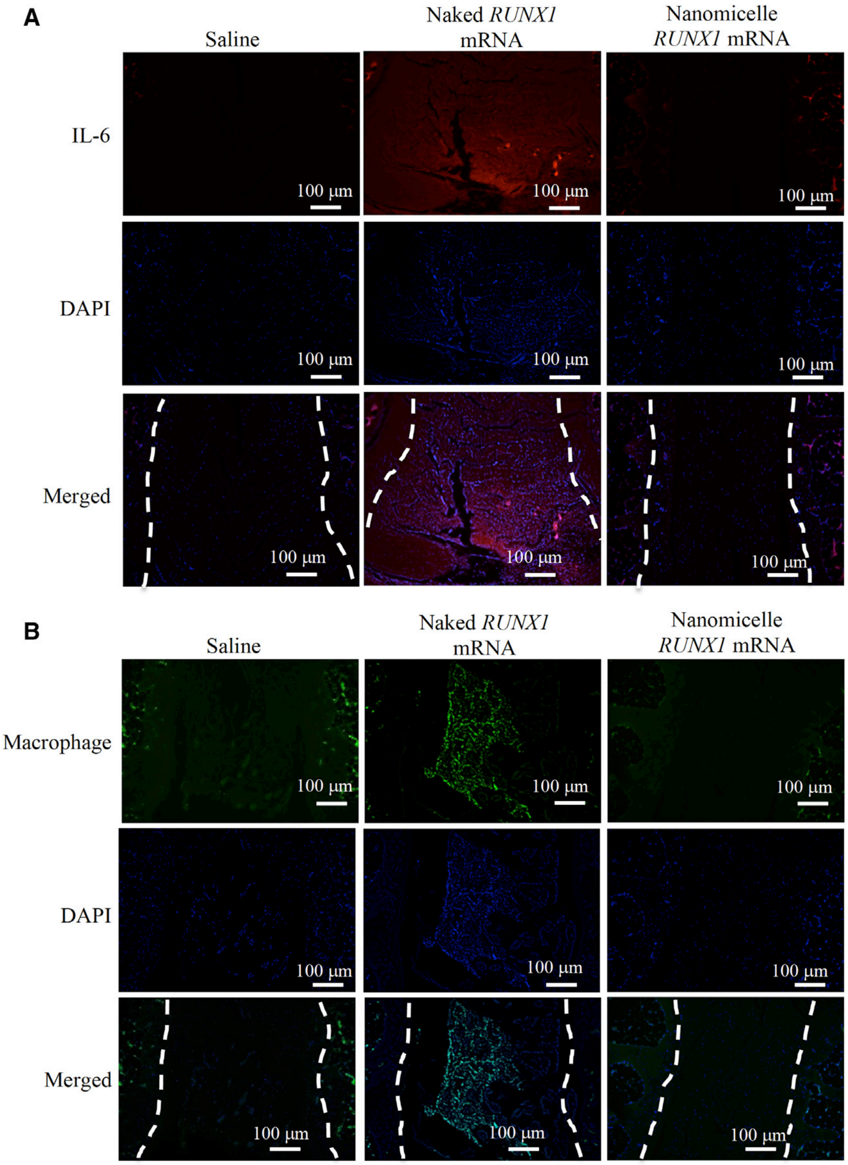

Figure 7. Immunohistochemical Staining for IL-6 and Macrophages

Sections were obtained 1 day after the administration of *RUNX1* mRNA nanomicelles, naked *RUNX1* mRNA, and saline. (A) IL-6 staining. The red signals represent the Alexa-647-labeled IL-6. (B) Macrophage marker staining. The green signals represent the Alexa 488-labeled macrophages. Dotted lines indicate the disk area (n = 3). Scale bar, 100 μm.

mRNA medicine is a promising alternative by directly using the residing cells in the disk. Considering the fact that the number of residing cells in the disk would be significantly decreased in the late stage of disk degeneration, mRNA medicine may be preferentially indicated for earlier stages of disk degeneration.

Of course, prior to considering the clinical application of the mRNA medicine for IVD diseases, there are some critical issues to be addressed. Safety is the most important issue. The transient manner of protein expression from the mRNA can be confirmed, but no one knows the long-term outcomes after the administration of transcription factor using mRNA. Much more information on the basis of clinical experience with mRNA medicine, including mRNA vaccines, many of which are now under clinical trials, is required for assuming the long-term safety. In addition, the administration route should be discussed. Unlike joint cartilage, the IVD requires an invasive approach owing to the deep location. The injection technique into the IVD is clinically established for the case such as discography, although a fluoroscopic monitoring is usually required for identifying the disk location. Nevertheless, this proof-of-concept study revealed that mRNA medicine has a potential for treating IVD degenerative diseases by introducing a cartilage-anabolic transcription factor into the host cells. More importantly, this approach will allow us to apply basic biological findings on disease-protective or tissue-regenerating factors for the treatment of degenerative diseases, opening a new era of drug development.

MATERIALS AND METHODS

In Vitro Transcription to Prepare the Therapeutic mRNA

RUNX1-FLAG mRNA was prepared using the pSP73-RUNX1-FL vector.[11] Briefly, the Runx1-FL sequence was sub-cloned into pSP73 vector, containing a 120-bp chemically synthesized poly(d(A/T) fragment at the downstream of the cDNA region[45] (Figure S1B). Then, the vectors were linearized with SpeI (Figure S1C), blunted with *E. coli* DNA polymerase I, purified with gel electrophoresis, and used as templates for *in vitro* transcription (IVT), using the mMESSAGE mMACHINE T7 Ultra Kit (Ambion, Invitrogen, Carlsbad, CA) to generate mRNA encoding FLAG and Runx1 fusion protein. The mRNA encoding Luc2 was similarly constructed from a vector encoding photinus pyralis luciferase (pGL4; Promega). Prior to the experiments, all transcribed mRNAs were purified by RNeasy mini kit (QIAGEN) and analyzed for size and purity with the Agilent RNA 6000 Nano Assay on a BioAnalyzer 2100 (Agilent Technologies) (Figure S1C).

Preparation of PEGylated mRNA Nanomicelles

A PEGylated block catiomer was previously developed[21] through a simple and affordable synthetic procedure based on an aminolysis of benzyl groups in the side chain of the poly(β-benzyl L-aspartate)

(PBLA) segment of PEG-PBLA block copolymer, with diethylenetriamine (DET) to generate N-substituted polyaspartamides (Figure S1A) bearing 2 aminoethylene repeats in the side chain. The block catiomer thus prepared (PEG [molecular weight (MW) = 42,000]-poly{*N*-[*N*′-(2-aminoethyl)-2-aminoethyl]aspartamide}) was abbreviated as 42K-PEG-PAsp(DET). The degree of polymerization of the PAsp(DET) segment was determined to be 56 by ^{1}H NMR analysis, respectively. To prepare the mRNA nanomicelles, 42K-PEG-PAsp(DET) and mRNA were separately dissolved in 2-[4-(2-hydroxyethyl)piperazin-1-yl]ethanesulfonic acid (HEPES) buffer and mixed at a volume ratio of 1:2 (Figure S1A). The concentration of mRNA was set to 666 ng/μL, and that of 42K-PEG-PAsp(DET) was adjusted to keep a residual molar ratio of amino groups in 42K-PEG-PAsp(DET) to phosphate groups in mRNA (N:P ratio) of 3. The mRNA nanomicelles were revealed to have the diameter around 50 nm with almost neutral surface charge.[11] mRNA quantity was adjusted to be 4 μg in a 6 μL total transfection volume.

Rat Coccygeal Disk Puncture Model and Nanomicelle Administration

All animal experiments were approved by the Institutional Animal Care and Use Committee (IACUC) of the Innovation Center of NanoMedicine, Kawasaki Institute of Industrial Promotion. The 10- to 12-week-old male Sprague-Dawley rats (Charles River Laboratories, Japan) were anesthetized by inhalation of 2.5% isoflurane (Abbott) and placed in a prone position on the stereotaxic instrument (Narishige Group). A 1.0- to 1.5-cm sagittal incision was made on the tail to expose the coccygeal disk co4-5, and the surgery area was treated with 0.1% adrenalin, which largely ceased bleeding. Under microscope (Figure S9A), the rat coccygeal disk co4-5 was punctured transversally with a 20G needle, completely penetrating the fibrosus ring, and subsequently injected with 6 μL gene mixture, using a 30G micro-injection needle at 1 μL/min and 1-mm depth (Figure S9B), through the larger track created by the previous 20G needle puncture (Figure S9C). Under microscopic operation, we could confirm the gene mixture delivered by the 30G needle reached the disk NP area.

Luminescence Measurement to Examine PEGylated Nanomicelle-Mediated mRNA Expression

To measure *in vivo* firefly Luc2 expression, D-Luciferin substrate (Promega) was dissolved in PBS and adjusted to a final concentration of 15 mg/mL. Bioluminescence was measured by an IVIS imaging system (Xenogen) 5 min after intraperitoneally injecting 800 μL D-Luciferin substrate. Bioluminescent signals in the coccygeal disk co4-5 region were analyzed by background subtraction using Living Image Software (Xenogen).

Radiographic Images to Evaluate the Coccygeal Disk Shrinkage

Rats were anesthetized at 0, 2, and 4 weeks post-injury by intramuscular injection of Zoletil 50/Rompon and placed in a prone position in the radiographic instrument. X-ray images were captured at a distance of 60 cm from the tail (40 kVp, 5 mAs), and images were used for the evaluation of DHI at post-injection. The percent DHI was represented as post-puncture DHI/pre-puncture DHI × 100%, as Figure 2B shows.[17]

Coccygeal Disk Hydration Content Evaluated by MRI

MRI scans were performed using a 7T MR scanning system (Bruker BioSpin, Germany) at the Central Institute for Experimental Animals. T2-weighted sagittal sections were rendered using the following settings: fast spin echo sequence with a time to repetition of 2,000 ms and time to echo of 72 ms; slice thickness, 1 mm; interslice gap, 1 mm; matrix, 256; field of view, 60 mm; and number of averages, 2. A 60-mm volume resonator and a 2-cm diameter surface receive coil were used to maximize image resolution and quality.

Histological Examination

Rats were sacrificed at 2 and 4 weeks post-injury; coccygeal disks co4-5 were removed, fixed with 4% paraformaldehyde (PFA) in PBS, decalcified in 0.5 M EDTA for 2 weeks, embedded in paraffin, and serial sectioned in 5-μm thickness for H&E staining. Sagittal serial sections in the mid-zone of the vertebral body were prepared for immunohistochemical (IHC) staining by standard protocol. Briefly, slides were de-paraffined, washed, blocked, and immunostained with type II collagen (COL2A1 [M2139]) (sc-52658), aggrecan (H-300) (sc-25674), IL-6 (M-19) (sc-1265), and macrophage marker (MAC387) (sc-66204) primary antibodies (Santa Cruz Biotechnology) at 4°C overnight; then they were stained with Alexa 488- or Alexa 647-conjugated secondary antibody (Jackson ImmunoResearch Laboratories) at room temperature for 1 h and subsequently counterstained with DAPI (VECTASHIELD, VECTOR Laboratories) and observed by fluorescence microscope (Carl Zeiss).[46] For histochemical staining, the de-paraffined sections were rehydrated and stained with 3% Alcian blue (in glacial acetic acid) for 30 min, followed by dehydration with 100% ethanol, or incubated with antibodies to FLAG (F1804, M2; Sigma-Aldrich, St. Louis, MO), and visualized by the reaction of peroxidase and diaminobenzodine (DAB).

Statistical Analysis

Statistical comparisons were performed by two-way ANOVA and p values < 0.05 were considered significant. All *in vivo* data are representative of at least 4 independent experiments as indicated.

SUPPLEMENTAL INFORMATION

Supplemental Information can be found with this article online at https://doi.org/10.1016/j.omtn.2019.02.012.

AUTHOR CONTRIBUTIONS

C.-Y.L., S.T.C., S.U., and Y.K. conducted the experiments. K.K. and K.I. were involved in the planning and supervised the work, and they provided financial support and experimental facilities. C.-Y.L. and K.I. designed the experiments, processed the experimental data, performed the analysis, designed the figures, drafted the manuscript, and wrote the paper.

CONFLICTS OF INTEREST

There are no conflicts of interest.

ACKNOWLEDGMENTS

We thank Dr. Shinsuke Ohba, The University of Tokyo, for technical assistance with preparing the RUNX1-FL vector. This work was financially supported by JSPS KAKENHI grant JP15H03017, Center of Innovation (COI) Program (Center of Open Innovation Network for Smart Health) from the Japan Science and Technology Agency (JST), JSPS Core-to-Core Program, A. Advanced Research Networks, JSPS Postdoctoral Fellowship for Foreign Researchers (grant 26·04406, C.-Y.L. and K.I.), and Projects from Ministry of Science and Technology (MOST) and China Medical University (CMU), Taiwan (grants MOST 106-2314-B-039-053, MOST 107-2314-B-039-023, MOST 107-2622-E-039-003, and CMU106-N-18). We thank Sae Suzuki, Satomi Ogura, Asuka Miyoshi, and Katsue Morii (The University of Tokyo) for technical assistance with animal studies.

REFERENCES

1. Cheung, K.M., Karppinen, J., Chan, D., Ho, D.W., Song, Y.Q., Sham, P., Cheah, K.S., Leong, J.C., and Luk, K.D. (2009). Prevalence and pattern of lumbar magnetic resonance imaging changes in a population study of one thousand forty-three individuals. Spine *34*, 934–940.
2. Takatalo, J., Karppinen, J., Niinimäki, J., Taimela, S., Näyhä, S., Mutanen, P., Sequeiros, R.B., Kyllönen, E., and Tervonen, O. (2011). Does lumbar disc degeneration on magnetic resonance imaging associate with low back symptom severity in young Finnish adults? Spine *36*, 2180–2189.
3. Pattappa, G., Li, Z., Peroglio, M., Wismer, N., Alini, M., and Grad, S. (2012). Diversity of intervertebral disc cells: phenotype and function. J. Anat. *221*, 480–496.
4. Fontana, G., See, E., and Pandit, A. (2015). Current trends in biologics delivery to restore intervertebral disc anabolism. Adv. Drug Deliv. Rev. *84*, 146–158.
5. Grunhagen, T., Wilde, G., Soukane, D.M., Shirazi-Adl, S.A., and Urban, J.P. (2006). Nutrient supply and intervertebral disc metabolism. J. Bone Joint Surg. Am. *88* (*Suppl 2*), 30–35.
6. Urban, J.P., and Roberts, S. (2003). Degeneration of the intervertebral disc. Arthritis Res. Ther. *5*, 120–130.
7. Chan, D., Song, Y., Sham, P., and Cheung, K.M. (2006). Genetics of disc degeneration. Eur. Spine J. *15* (*Suppl 3*), S317–S325.
8. Sakai, D., and Andersson, G.B. (2015). Stem cell therapy for intervertebral disc regeneration: obstacles and solutions. Nat. Rev. Rheumatol. *11*, 243–256.
9. Mochida, J., Nishimura, K., Nomura, T., Toh, E., and Chiba, M. (1996). The importance of preserving disc structure in surgical approaches to lumbar disc herniation. Spine (Phila Pa 1976) *21*, 1556–1563.
10. Blanco, F.J., and Ruiz-Romero, C. (2013). New targets for disease modifying osteoarthritis drugs: chondrogenesis and Runx1. Ann. Rheum. Dis. *72*, 631–634.
11. Aini, H., Itaka, K., Fujisawa, A., Uchida, H., Uchida, S., Fukushima, S., Kataoka, K., Saito, T., Chung, U.I., and Ohba, S. (2016). Messenger RNA delivery of a cartilage-anabolic transcription factor as a disease-modifying strategy for osteoarthritis treatment. Sci. Rep. *6*, 18743.
12. Jirikowski, G.F., Sanna, P.P., Maciejewski-Lenoir, D., and Bloom, F.E. (1992). Reversal of diabetes insipidus in Brattleboro rats: intrahypothalamic injection of vasopressin mRNA. Science *255*, 996–998.
13. Boczkowski, D., Nair, S.K., Snyder, D., and Gilboa, E. (1996). Dendritic cells pulsed with RNA are potent antigen-presenting cells in vitro and in vivo. J. Exp. Med. *184*, 465–472.
14. Zou, S., Scarfo, K., Nantz, M.H., and Hecker, J.G. (2010). Lipid-mediated delivery of RNA is more efficient than delivery of DNA in non-dividing cells. Int. J. Pharm. *389*, 232–243.
15. Lin, C.Y., Perche, F., Ikegami, M., Uchida, S., Kataoka, K., and Itaka, K. (2016). Messenger RNA-based therapeutics for brain diseases: An animal study for augmenting clearance of beta-amyloid by intracerebral administration of neprilysin mRNA loaded in polyplex nanomicelles. J. Control. Release *235*, 268–275.
16. Liao, J.C. (2016). Cell Therapy Using Bone Marrow-Derived Stem Cell Overexpressing BMP-7 for Degenerative Discs in a Rat Tail Disc Model. Int. J. Mol. Sci. *17*, E147.
17. Inoue, H., Montgomery, S.R., Aghdasi, B., Kaner, T., Tan, Y., Tian, H., Terrell, R., Wang, J.C., and Daubs, M.D. (2015). The effect of bone morphogenetic protein-2 injection at different time points on intervertebral disk degeneration in a rat tail model. J. Spinal Disord. Tech. *28*, E35–E44.
18. Grunert, P., Hudson, K.D., Macielak, M.R., Aronowitz, E., Borde, B.H., Alimi, M., Njoku, I., Ballon, D., Tsiouris, A.J., Bonassar, L.J., and Härtl, R. (2014). Assessment of intervertebral disc degeneration based on quantitative magnetic resonance imaging analysis: an in vivo study. Spine *39*, E369–E378.
19. Uchida, S., Hayakawa, K., Ogata, T., Tanaka, S., Kataoka, K., and Itaka, K. (2016). Treatment of spinal cord injury by an advanced cell transplantation technology using brain-derived neurotrophic factor-transfected mesenchymal stem cell spheroids. Biomaterials *109*, 1–11.
20. Baba, M., Itaka, K., Kondo, K., Yamasoba, T., and Kataoka, K. (2015). Treatment of neurological disorders by introducing mRNA in vivo using polyplex nanomicelles. J. Control. Release *201*, 41–48.
21. Uchida, S., Itaka, K., Uchida, H., Hayakawa, K., Ogata, T., Ishii, T., Fukushima, S., Osada, K., and Kataoka, K. (2013). In vivo messenger RNA introduction into the central nervous system using polyplex nanomicelle. PLoS ONE *8*, e56220.
22. Johnson, K., Zhu, S., Tremblay, M.S., Payette, J.N., Wang, J., Bouchez, L.C., Meeusen, S., Althage, A., Cho, C.Y., Wu, X., and Schultz, P.G. (2012). A stem cell-based approach to cartilage repair. Science *336*, 717–721.
23. Yano, F., Hojo, H., Ohba, S., Fukai, A., Hosaka, Y., Ikeda, T., Saito, T., Hirata, M., Chikuda, H., Takato, T., et al. (2013). A novel disease-modifying osteoarthritis drug candidate targeting Runx1. Ann. Rheum. Dis. *72*, 748–753.
24. Wang, Y., Belflower, R.M., Dong, Y.F., Schwarz, E.M., O'Keefe, R.J., and Drissi, H. (2005). Runx1/AML1/Cbfa2 mediates onset of mesenchymal cell differentiation toward chondrogenesis. J. Bone Miner. Res. *20*, 1624–1636.
25. Wotton, S., Terry, A., Kilbey, A., Jenkins, A., Herzyk, P., Cameron, E., and Neil, J.C. (2008). Gene array analysis reveals a common Runx transcriptional programme controlling cell adhesion and survival. Oncogene *27*, 5856–5866.
26. Komori, T. (2005). Regulation of skeletal development by the Runx family of transcription factors. J. Cell. Biochem. *95*, 445–453.
27. Sato, S., Kimura, A., Ozdemir, J., Asou, Y., Miyazaki, M., Jinno, T., Ae, K., Liu, X., Osaki, M., Takeuchi, Y., et al. (2008). The distinct role of the Runx proteins in chondrocyte differentiation and intervertebral disc degeneration: findings in murine models and in human disease. Arthritis Rheum. *58*, 2764–2775.
28. Sahin, U., Karikó, K., and Türeci, Ö. (2014). mRNA-based therapeutics–developing a new class of drugs. Nat. Rev. Drug Discov. *13*, 759–780.
29. Li, J., Guan, H., Liu, H., Zhao, L., Li, L., Zhang, Y., Tan, P., Mi, B., and Li, F. (2017). Epoxyeicosanoids prevent intervertebral disc degeneration in vitro and in vivo. Oncotarget *8*, 3781–3797.
30. Zangi, L., Lui, K.O., von Gise, A., Ma, Q., Ebina, W., Ptaszek, L.M., Später, D., Xu, H., Tabebordbar, M., Gorbatov, R., et al. (2013). Modified mRNA directs the fate of heart progenitor cells and induces vascular regeneration after myocardial infarction. Nat. Biotechnol. *31*, 898–907.
31. Huang, C.-L., Leblond, A.-L., Turner, E.C., Kumar, A.H., Martin, K., Whelan, D., O'Sullivan, D.M., and Caplice, N.M. (2015). Synthetic chemically modified mrna-based delivery of cytoprotective factor promotes early cardiomyocyte survival post-acute myocardial infarction. Mol. Pharm. *12*, 991–996.
32. Perche, F., Uchida, S., Akiba, H., Lin, C.Y., Ikegami, M., Dirisala, A., Nakashima, T., Itaka, K., Tsumoto, K., and Kataoka, K. (2017). Improved Brain Expression of Anti-Amyloid β scFv by Complexation of mRNA Including a Secretion Sequence with PEG-based Block Catiomer. Curr. Alzheimer Res. *14*, 295–302.
33. Baek, S., Oh, J., Song, J., Choi, H., Yoo, J., Park, G.-Y., Han, J., Chang, Y., Park, H., Kim, H., et al. (2017). Generation of Integration-Free Induced Neurons Using Graphene Oxide-Polyethylenimine. Small *13*, 1601993.

34. Matsui, A., Uchida, S., Ishii, T., Itaka, K., and Kataoka, K. (2015). Messenger RNA-based therapeutics for the treatment of apoptosis-associated diseases. Sci. Rep. *5*, 15810.

35. Nabhan, J.F., Wood, K.M., Rao, V.P., Morin, J., Bhamidipaty, S., LaBranche, T.P., Gooch, R.L., Bozal, F., Bulawa, C.E., and Guild, B.C. (2016). Intrathecal delivery of frataxin mRNA encapsulated in lipid nanoparticles to dorsal root ganglia as a potential therapeutic for Friedreich's ataxia. Sci. Rep. *6*, 20019.

36. Roseman, D.S., Khan, T., Rajas, F., Jun, L.S., Asrani, K.H., Isaacs, C., Farelli, J.D., and Subramanian, R.R. (2018). G6PC mRNA Therapy Positively Regulates Fasting Blood Glucose and Decreases Liver Abnormalities in a Mouse Model of Glycogen Storage Disease 1a. Mol. Ther. *26*, 814–821.

37. Balmayor, E.R., Geiger, J.P., Aneja, M.K., Berezhanskyy, T., Utzinger, M., Mykhaylyk, O., Rudolph, C., and Plank, C. (2016). Chemically modified RNA induces osteogenesis of stem cells and human tissue explants as well as accelerates bone healing in rats. Biomaterials *87*, 131–146.

38. Khorsand, B., Elangovan, S., Hong, L., Dewerth, A., Kormann, M.S.D., and Salem, A.K. (2017). A Comparative Study of the Bone Regenerative Effect of Chemically Modified RNA Encoding BMP-2 or BMP-9. AAPS J. *19*, 438–446.

39. Groth, K., Berezhanskyy, T., Aneja, M.K., Geiger, J., Schweizer, M., Maucksch, L., Pasewald, T., Brill, T., Tigani, B., Weber, E., et al. (2017). Tendon healing induced by chemically modified mRNAs. Eur. Cell. Mater. *33*, 294–307.

40. Yang, Q., Xu, H.W., Hurday, S., and Xu, B.S. (2016). Construction Strategy and Progress of Whole Intervertebral Disc Tissue Engineering. Orthop. Surg. *8*, 11–18.

41. Nukaga, T., Sakai, D., Tanaka, M., Hiyama, A., Nakai, T., and Mochida, J. (2016). Transplantation of activated nucleus pulposus cells after cryopreservation: efficacy study in a canine disc degeneration model. Eur. Cell. Mater. *31*, 95–106.

42. Mochida, J., Sakai, D., Nakamura, Y., Watanabe, T., Yamamoto, Y., and Kato, S. (2015). Intervertebral disc repair with activated nucleus pulposus cell transplantation: a three-year, prospective clinical study of its safety. Eur. Cell. Mater. *29*, 202–212.

43. Kandel, R., Roberts, S., and Urban, J.P. (2008). Tissue engineering and the intervertebral disc: the challenges. Eur. Spine J. *17* (*Suppl 4*), 480–491.

44. Hohaus, C., Ganey, T.M., Minkus, Y., and Meisel, H.J. (2008). Cell transplantation in lumbar spine disc degeneration disease. Eur. Spine J. *17* (*Suppl 4*), 492–503.

45. Holtkamp, S., Kreiter, S., Selmi, A., Simon, P., Koslowski, M., Huber, C., Türeci, O., and Sahin, U. (2006). Modification of antigen-encoding RNA increases stability, translational efficacy, and T-cell stimulatory capacity of dendritic cells. Blood *108*, 4009–4017.

46. Derbigny, W.A., Shobe, L.R., Kamran, J.C., Toomey, K.S., and Ofner, S. (2012). Identifying a role for Toll-like receptor 3 in the innate immune response to Chlamydia muridarum infection in murine oviduct epithelial cells. Infect. Immun. *80*, 254–265.

OMTN, Volume *16*

Supplemental Information

Treatment of Intervertebral Disk Disease by the Administration of mRNA Encoding a Cartilage-Anabolic Transcription Factor

Chin-Yu Lin, Samuel Thomas Crowley, Satoshi Uchida, Yuji Komaki, Kazunori Kataoka, and Keiji Itaka

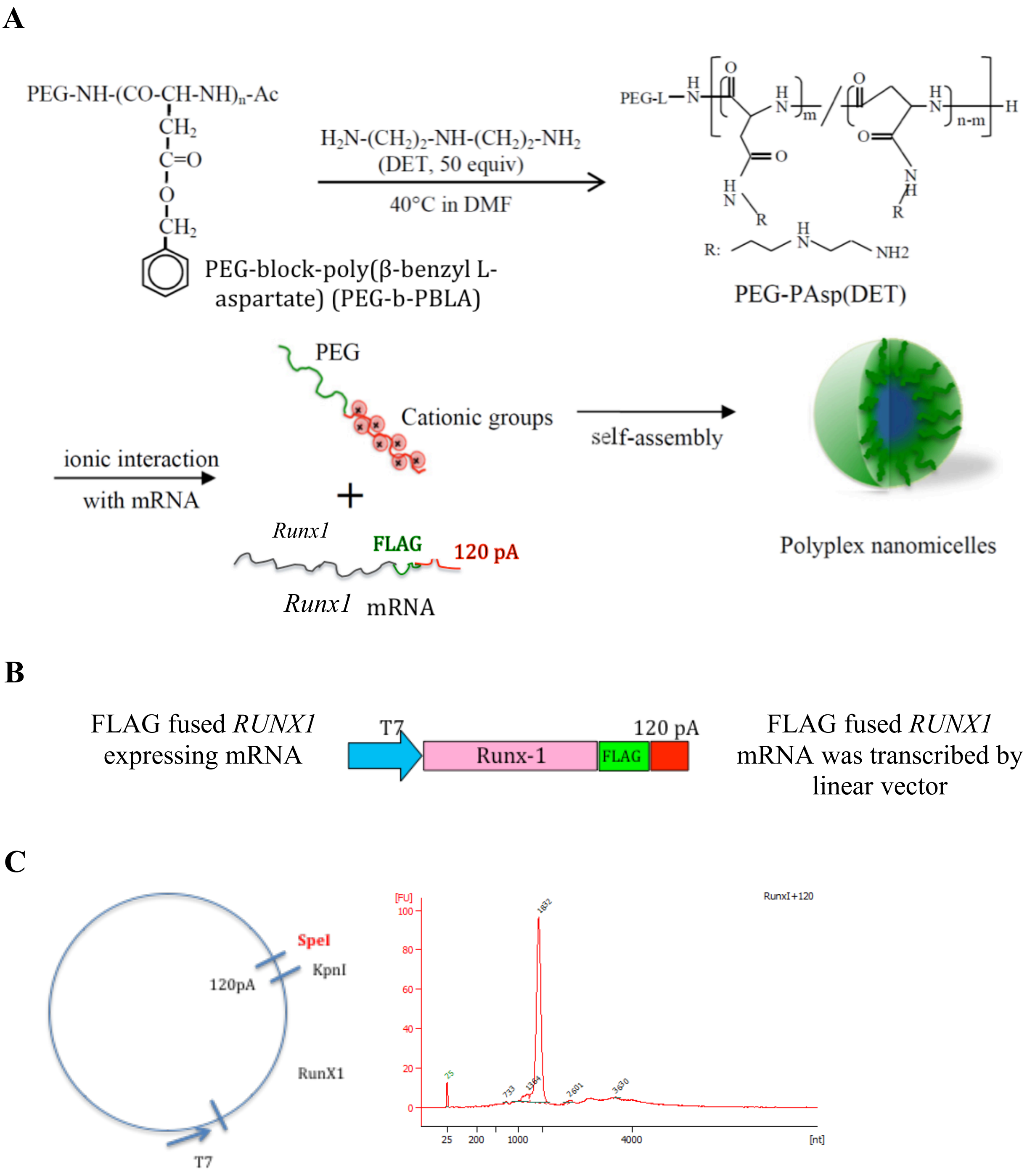

Fig. S1. (A) PEG- Poly{N-[N'-(2-aminoethyl)-2-aminoethyl]aspartamide} block copolymer (PEG-PAsp[DET]) was synthesized and self-assembled with mRNA to form polyplex nanomicelles. (B) The Runx1-FL sequence was sub-cloned into a pSP73 vector, containing a 120 bp chemically synthesized poly(d(A/T) fragment downstream of the cDNA region. (C) The vectors were linearized with SpeI for IVT, and all transcribed mRNAs were analyzed for size and purity with the Agilent RNA 6000 Nano Assay on a BioAnalyzer 2100.

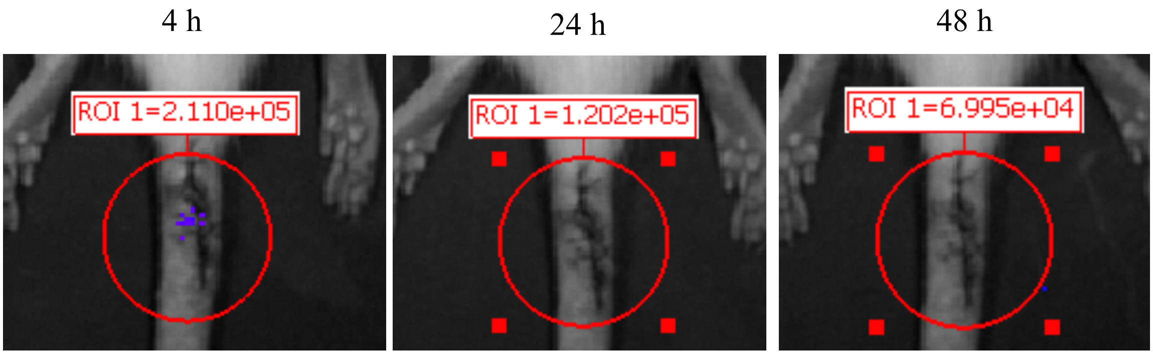

Fig. S2. IVIS images of *Luc* mRNA in the form of naked mRNA without nanomicelles.

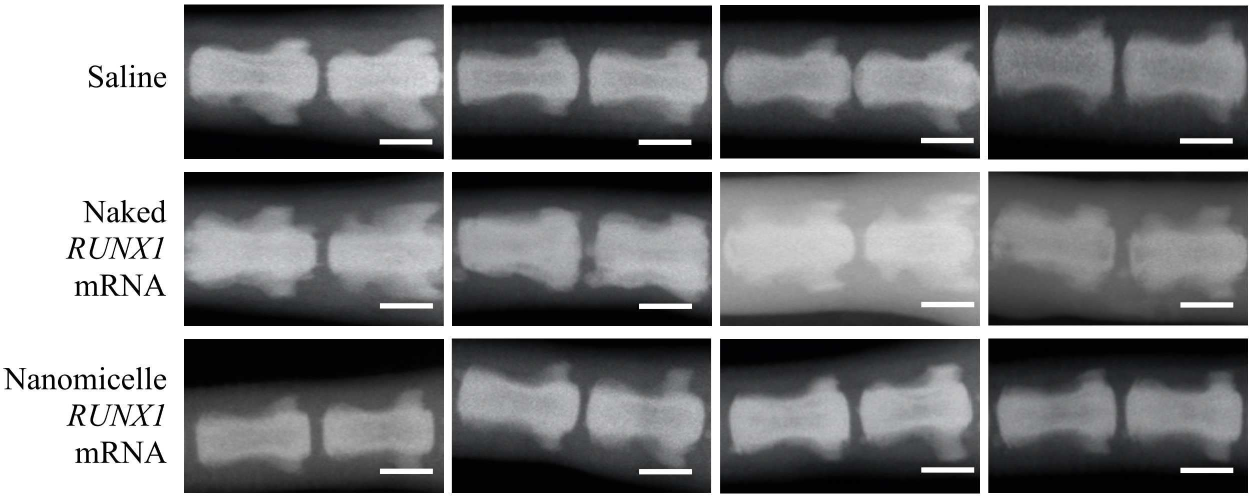

Fig. S3. All other radiographic images of coccygeal disk at 4 weeks post-injection (bar= 4 mm).

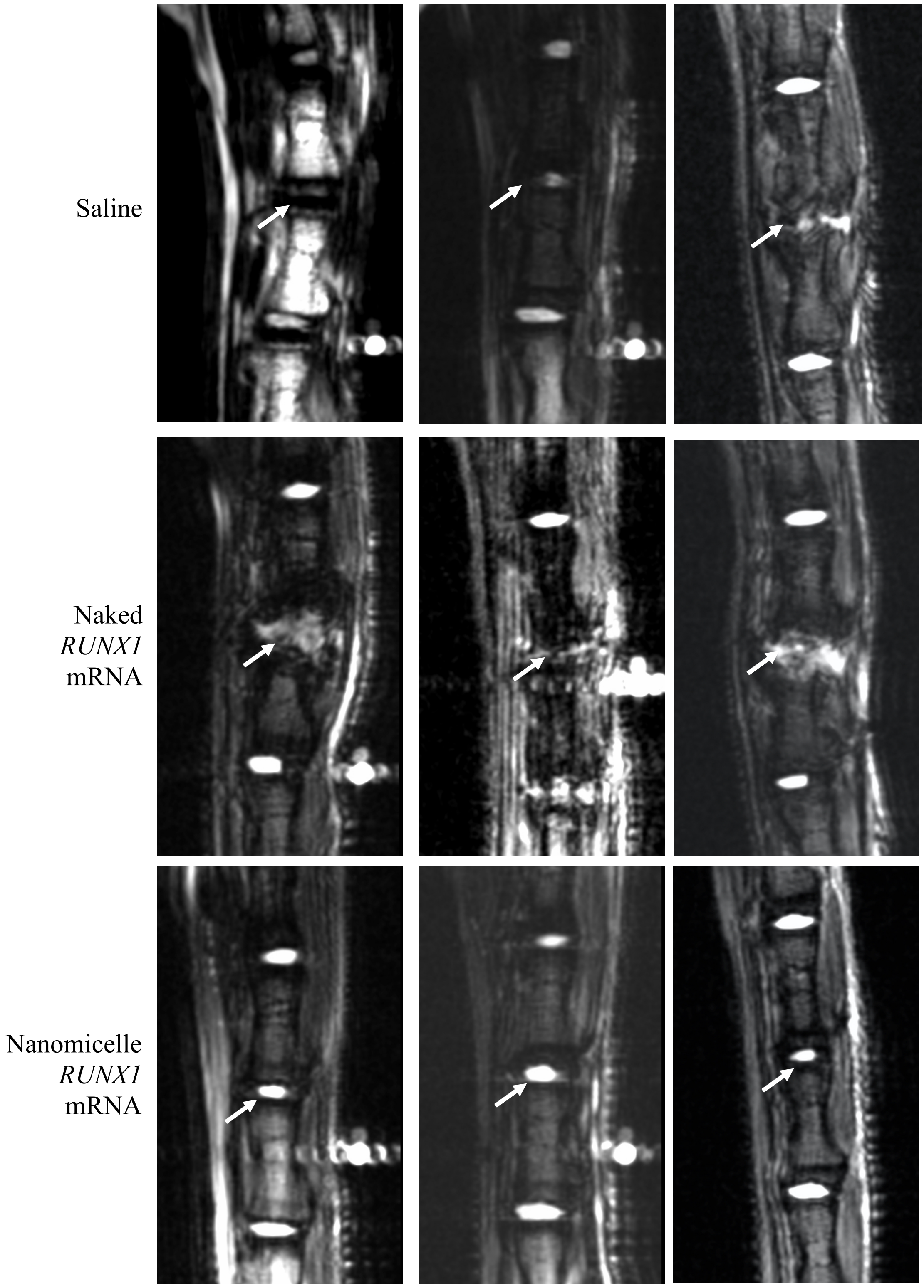

Fig. S4. All other MRI-T2 images of coccygeal disk at 4 weeks post-injection. Arrows indicate the micro-injected disks.

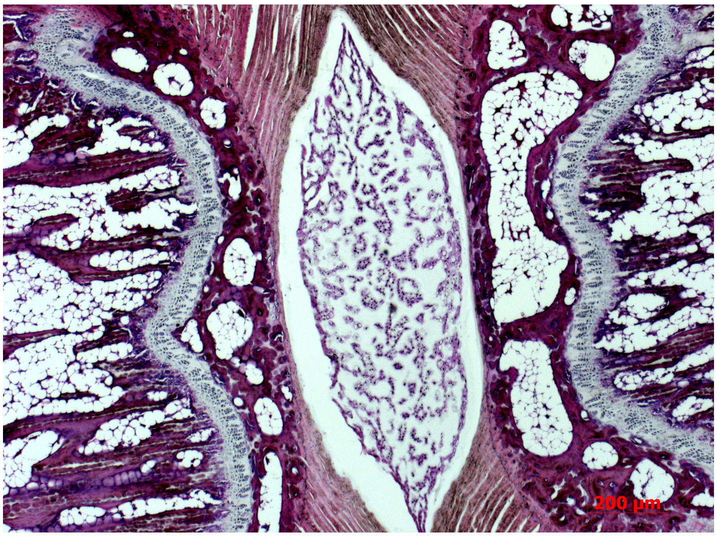

Fig. S5. H&E staining of Intact disk.

Collagen type II

Aggrecan

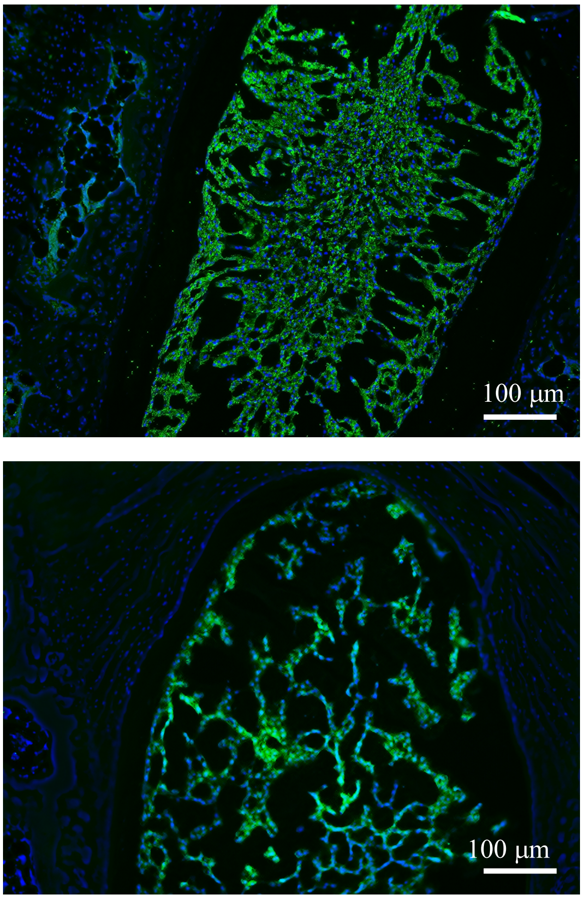

Fig. S6. Type II Collagen and aggrecan IHC of Intact disk.

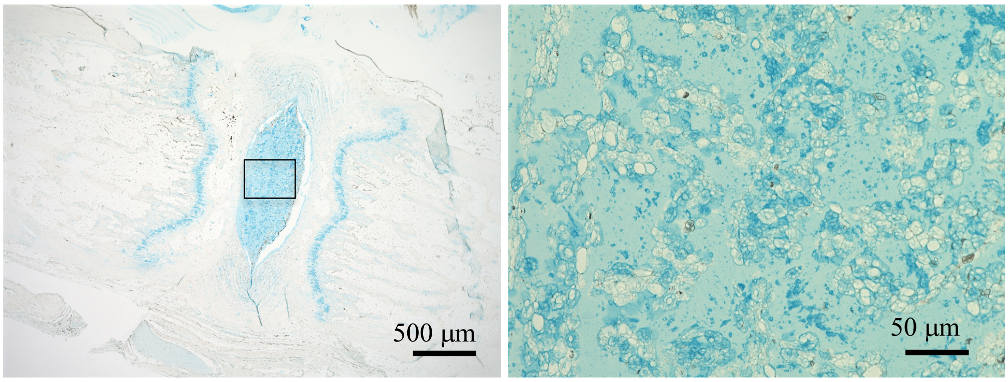

Fig. S7. Alcian blue staining of intact disk.

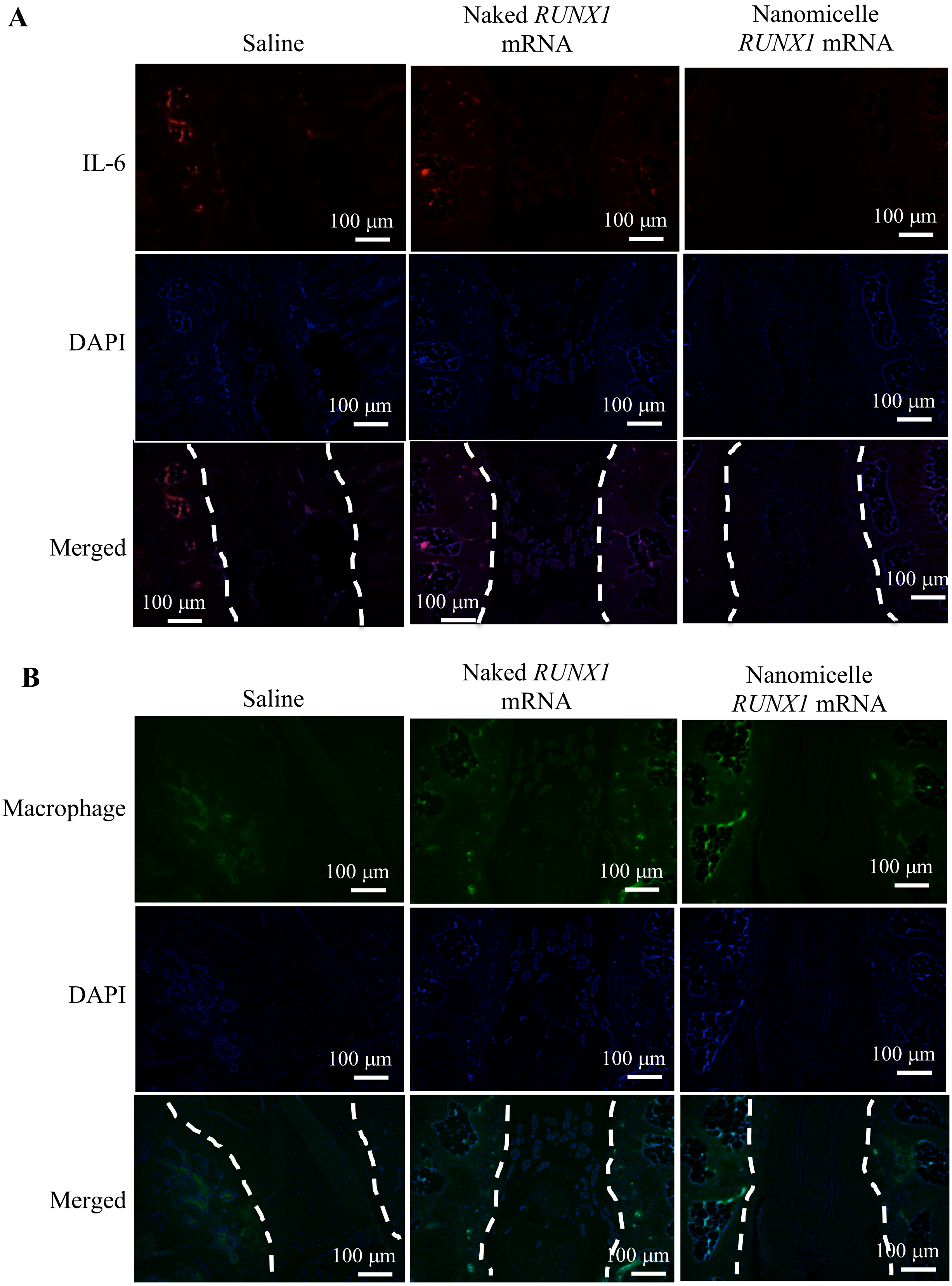

Fig. S8. **IL-6 and macrophage IHC staining of disks removed at 2 weeks post-injection. (A)** IL-6 IHC, the 2nd Ab was labeled with Alexa-647. **(B)** Macrophage IHC, the 2nd Ab was labeled with Alexa-488. Dotted lines indicate the disk area. Bar = 100 μm.

A

B

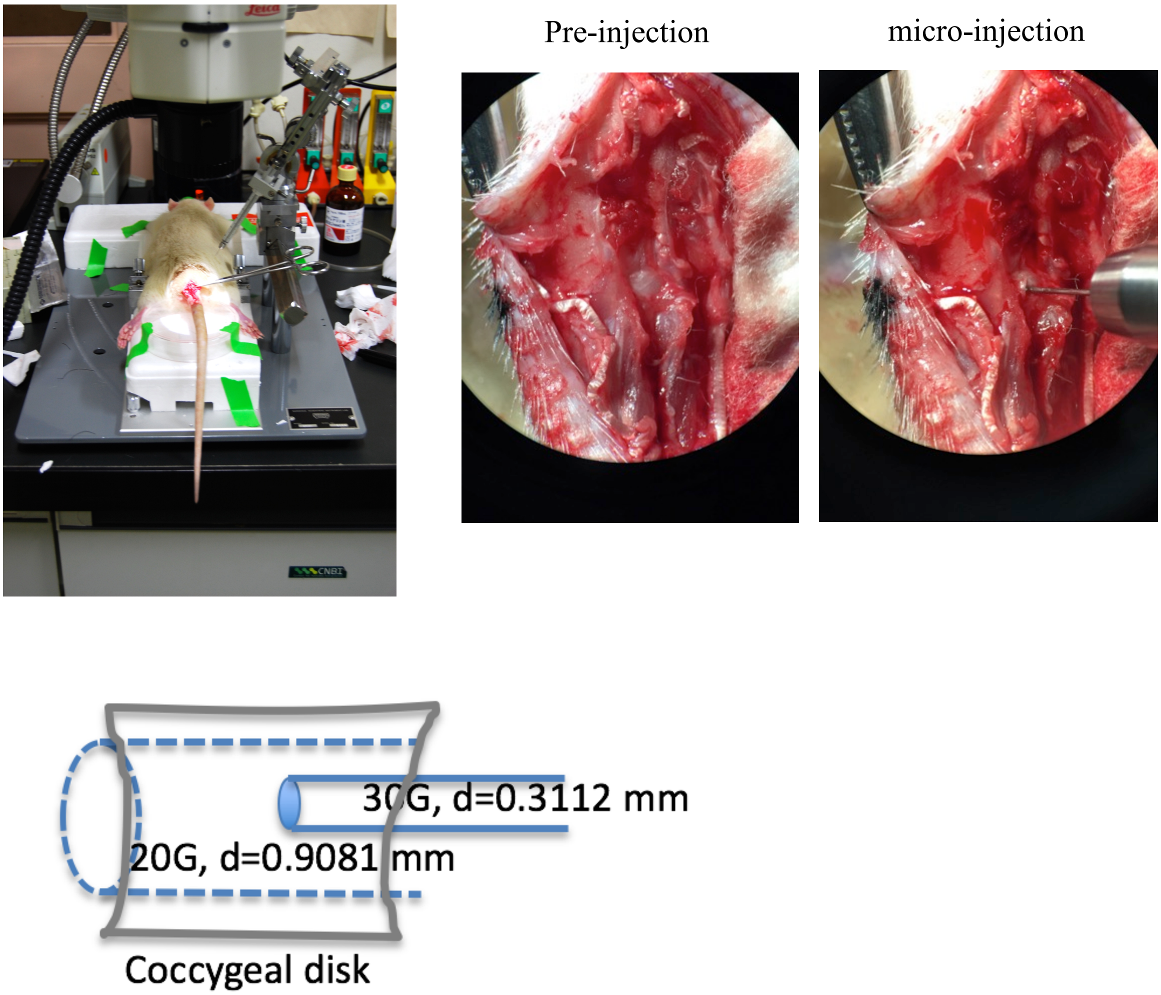

C

Fig. S9. (A) Rat coccygeal disks were operated on under the assistance of a stereotaxic instrument and microscope. (B) Gene mixtures were micro-injected with 30G micro-injection needle at 1 μl/min at a 1 mm depth. (C) Illustration of disk punctured with 20G needle, subsequently micro-injected with 30G needle through the larger track.
